# Geographic accessibility to public and private health facilities in Kenya in 2021: An updated geocoded inventory and spatial analysis

**DOI:** 10.3389/fpubh.2022.1002975

**Published:** 2022-11-03

**Authors:** Angela K. Moturi, Laurissa Suiyanka, Eda Mumo, Robert W. Snow, Emelda A. Okiro, Peter M. Macharia

**Affiliations:** ^1^Population Health Unit, Kenya Medical Research Institute-Wellcome Trust Research Programme, Nairobi, Kenya; ^2^Nuffield Department of Medicine, Centre for Tropical Medicine and Global Health, University of Oxford, Oxford, United Kingdom; ^3^Centre for Health Informatics, Computing, and Statistics, Lancaster Medical School, Lancaster University, Lancaster, United Kingdom

**Keywords:** spatial access, health facility, private sector, public sector, travel time, disease prevalence, inequalities, universal health access

## Abstract

**Objectives:**

To achieve universal health coverage, adequate geographic access to quality healthcare services is vital and should be characterized periodically to support planning. However, in Kenya, previous assessments of geographic accessibility have relied on public health facility lists only, assembled several years ago. Here, for the first time we assemble a geocoded list of public and private health facilities in 2021 and make use of this updated list to interrogate geographical accessibility to all health providers.

**Methods:**

Existing health provider lists in Kenya were accessed, merged, cleaned, harmonized, and assigned a unique geospatial location. The resultant master list was combined with road network, land use, topography, travel barriers and healthcare-seeking behavior within a geospatial framework to estimate travel time to the nearest (i) private, (ii) public, and (iii) both (public and private-PP) health facilities through a travel scenario involving walking, bicycling and motorized transport. The proportion of the population within 1 h and outside 2-h was computed at 300 × 300 spatial resolution and aggregated at subnational units used for decision-making. Areas with a high disease prevalence for common infections that were outside 1-h catchment (dual burden) were also identified to guide prioritization.

**Results:**

The combined database contained 13,579 health facilities, both in the public (55.5%) and private-for-profit sector (44.5%) in 2021. The private health facilities' distribution was skewed toward the urban counties. Nationally, average travel time to the nearest health facility was 130, 254, and 128 min while the population within 1-h was 89.4, 80.5, and 89.6% for the public, private and PP health facility, respectively. The population outside 2-h were 6% for public and PP and 11% for the private sector. Mean travel time across counties was heterogeneous, while the population within 1-h ranged between 38 and 100% in both the public sector and PP. Counties in northwest and southeast Kenya had a dual burden.

**Conclusion:**

Continuous updating and geocoding of health facilities will facilitate an improved understanding of healthcare gaps for planning. Heterogeneities in geographical access continue to persist, with some areas having a dual burden and should be prioritized toward reducing health inequities and attaining universal health coverage.

## Background

Key to achievement of Universal Health Coverage (UHC) within the Sustainable Development Goal (SDG) agenda is access to quality healthcare services (1). In sub-Saharan Africa (SSA), adequate access to healthcare is hampered by barriers such as financial limitations, poorly maintained and inaccessible roads, negative cultural beliefs, climate change and health disasters, inadequate health service providers and poor quality service provision (2, 3). While healthcare access is multi-dimensional, covering availability, acceptability, accommodation and affordability, geographic accessibility remains a major obstacle to UHC given the dynamic nature of the population in need of healthcare services relative to fixed service providers (4–6).

Geographic accessibility, the difficulty or ease in physically moving from the location where a need for health services is triggered to the service provider location, has been shown to influence health outcomes. For example, childhood immunization (7), institutional deliveries, childhood mortality (8–10), surgical care (11, 12), and maternal and newborn health (13–16) in SSA. Through spatial accessibility analyses, health coverage gaps have been identified and used as the basis for planning population health care and targeted resource allocation (17). Thus, the availability of quality health services within convenient physical proximity of the population remains an important health development goal in SSA.

Spatial accessibility has been estimated through modeling techniques or self-reported approaches (4) with the choice of a particular method driven by the objective, availability of data and health-seeking behavior. Regardless of the approach used to model geographic access, a fundamental requirement is an up-to-date, geocoded Master Health Facility List (MHFL), an inventory of health service providers and attributes of the services they offer. A key component of the MHFL is the spatial location of health service providers because it enables linked services, diseases and population catchments (18, 19). Accordingly, recent attempts have been made to ensure that geo-coded MHFLs are available in the public domain in SSA (12, 20–22). These existing spatial databases have been used with geospatial frameworks to characterize spatial accessibility to health service providers at different spatial scales (11, 12, 22–25).

The earliest formal metrics assessing physical access to healthcare providers at country level in Kenya was in 2003 (26) and later updated in 2008 (27). This is in addition to continental level analyses (11, 12, 22, 23) where Kenya was included and other analyses at a much smaller geographical scale (24, 25, 28). These spatial accessibility analyses have relied on data that was assembled several years ago (12, 20, 21, 29) and has not been updated to reflect recent changes in newly opened facilities, closed health facilities, changes in the designation of healthcare providers (such as upgrading level 2–3), improvement in geocoding techniques and data sources. More importantly all the previous attempts in Kenya suffer a significant drawback. They have only focused on services provided by governments and public sector partners and ignored the private sector. The private sector constitutes over 45% of all health facilities in Kenya (30), therefore, any health metric that ignores their contributed is likely to be flawed. Therefore, they should also be curated with consequent updating, geo-referencing and linking service provision to the population (12, 20–22).

To address these information gaps, we have undertaken a re-examination of the completeness, fidelity and geo-coding of the Kenyan health facilities providing preventive, diagnostic and curative services to the general population including both public and private facilities for the first time. We use this updated list to compute travel time to the nearest health facility, population covered with relevant time thresholds and highlight areas that have high disease infection prevalence and are located far from service providers to guide prioritization of resources. For policy relevance, estimates are summarized at the county and sub county levels which are the subnational units of decision-making (31).

## Methods

### Kenya context

The Republic of Kenya, a lower middle-income country, is located in East Africa and had a population of 47.6 million people in 2019 (32). Its human settlement pattern is heterogenous, with the highest densities in areas around Lake Victoria, the western and central region, and the coastal areas. In contrast, southern and northern areas are sparsely populated (Supplementary Figure 1). About a third (31%) of Kenya's population (14.8 million) in 2019 resided in urban areas with the counties of Mombasa, Nairobi, Kisumu, Machakos, Kiambu, Uasin Gishu, Nakuru, and Kajiado accounting for 70% of the urban population (32). These patterns impact health service delivery (infrastructure, demand, and planning) and the local burden of disease. Health provision comprises governmental, non-governmental, faith-based organizations, and private-for-profit managed health facilities. The health providers are structured hierarchically into tiers: community (community units), primary care (dispensaries, clinics, and health centers), county referral (first and second referral hospitals) and national referral (tertiary care hospitals).

With the promulgation of Kenya's new constitution in 2010 and after the 2013 general elections, two tiers of government were introduced: a national government and 47 semi-autonomous county governments that are now used for policy planning. With decentralization, county governments are mandated with ownership and management of county healthcare facilities (county hospitals, health centers and dispensaries) and healthcare service delivery. In contrast, the central government, through the Ministry of Health (MoH) manages national referral hospitals, health policy and regulatory functions. The Kenyan constitution enshrines the right to the highest achievable standard of health, including geographical access for all (31). Key priorities in health policy include improving access to essential primary health care and ensuring that high-quality health services are available to the population (33).

### Health facility mapping in Kenya

Mapping of health service providers in Kenya began before independence in the 1950 s, providing hand-drawn maps of government run health centers and hospitals (34). Forty years later, the significance of spatially defined health service provision was restituted (35). During the early 2000 s, Kenya maintained multiple health service provision lists (26) with the KEMRI-Wellcome Trust Research Programme (KWTRP) providing a collaborative service to the MoH, assisting in the geo-location of health services nationwide (12, 20, 26, 27, 36). In early 2000, KWTRP combined these multiple listings and manually geo-coded facilities using online place name gazetteers, hand-drawn maps in district development reports, topographical maps and occasionally from Global Positioning Systems (GPS) (26). With the recognition of the value of spatially configured data and the growing availability of place name gazetteers and GPS data, this process was repeated in 2008 (27), 2016 (20) and 2017 (36). However, all these geo-coded listings excluded the private-for-profit sector.

The generated facility spatial coordinates from some geo-coding efforts (26, 27) have been integrated into various versions of the Kenyan MHFL (37), although these coordinates are not publicly available. In addition, these coordinates are incomplete and have not all been verified since the initial geocoding mechanisms were rudimentary. Presently, under the devolved local governance in Kenya, county health authorities are responsible for maintaining and geocoding all county-level health facilities. Updated county-level facility coordinates are forwarded to the Health Information Unit of the MoH which updates the national MHFL. The online portal of the MHFL allows for a pinned visualization of facility locations on a web map for which coordinates have been assembled. Not all facilities on the county-level MHFL are available on the visualization tool, and there are no verification processes for facility coordinates.

In contrast, the sources of information used to geocode health facility locations have changed over time, from on-screen digitizing of hand-drawn and topographical district maps, and online digital place name gazetteers to increasing GPS-enabled devices such as smartphones to position facilities by national NGOs, research, and survey agencies. This has also been facilitated by improving the sources used to assign geographic coordinates to health service providers over the last decade. OpenStreetMap (OSM), the Wikipedia of maps, has grown because of voluntary mapping communities, governmental, non-governmental and humanitarian organizations that contribute to open geographic databases (38). This has been achieved through initiatives such as Humanitarian OpenStreetMap Team (HOT) Tasking Manager0F^1^, Missing Maps1F^2^ and YouthMappers2F^3^. Gazetteers collect and make available place names and their coordinates (39) increasing the quantity and quality of geocoded place-names (40). The detail and resolution of imagery available on Google Earth have improved significantly. After their update in 2017, 24 million satellite photos from the past 37 years have been embedded into a new layer of Google Earth, facilitated by the European Space Agency (ESA), the European Union's Copernicus programme, and its sentinel satellites, The National Aeronautics and Space Administration (NASA) and the US Geological Survey3F (41).

As changes were happening in Kenya's MHFL and increased geocoding platforms, in 2011, District Health Information System 2 (DHIS2) (42)—an open-source, web-based platform used as a health management information system was launched in Kenya (43). By 2016, DHIS2 replaced previously fragmented reporting systems and now serves as the single, harmonized health reporting platform for all surveillance systems. Information is available through MoH authorized access (42). However, geographic coordinates are not available within the DHIS2 platform for external users. MHFL and DHIS2 lack complete interoperability, however, there are continued efforts by MoH to harmonize the two.

### Data assembly

#### Health facilities

Health facilities are dynamic; new facilities are built to meet growing population demand; services close and existing services are repurposed to meet growing service demand. This dynamism and changes in geocoding platforms and fragmented non-harmonized coordinates prompted a re-examination of the Kenyan MHFL and linkage to the DHIS2 platform for health facilities providing preventive, diagnostic, and curative services to the general population both public and private.

##### Completeness and fidelity

The Kenyan MHFL and DHIS2 health facility data were accessed between April and August 2021. Data were first cleaned to standardize the naming and spelling of each facility. Facilities indicated as closed or non-operational in the MHFL were excluded. Data were linked between the MHFL and DHIS2 using the 5-digit MHFL code available in both data series resulting in a single harmonized database. Duplicates within the merged database were deleted, including annexes to main facilities sometimes included separately in the MHFL. Facilities not providing curative and diagnostic services to a wider general population were excluded and are summarized in Supplementary Table 1.

##### Geo-coding

For each facility on the merged DHIS2/MHFL database, coordinates were first checked against geo-coded databases maintained by KWTRP to attribute coordinates derived from GPS recordings uniquely. The locations of the remaining facilities were then sought using Google Earth based on structure names and ArcMap version 10.5 (ESRI Inc., Redlands, CA, USA) spatial overlays of first and second-level administrative boundaries (county, sub-county). Additional spatial overlays were used out to determine whether facilities were located in urban and non-urban areas based on a publicly available database that digitized extents of urban areas using satellite imagery based on the 2019 Kenya census listings (32, 44). For those that could not be identified by name on Google Earth, the visualization tool on the MHFL web portal was used to locate a physical structure in Google Earth and confirming the location extracted. Those where GPS coordinates, named locations or approximate locations on Google Earth were not available were left un-geo-coded.

#### Factors associated with travel

Spatial layers of factors that affect travel between residential areas and health facilities, including road network, land use, elevation, and travel barriers, were compiled. These spatial layers serve as inputs into geospatial framework of modeling travel time.

##### Road network

To account for the travel routes to health facilities, road network data was sourced from the Ministry of Transport of Kenya. This spatial layer employed gold standard GPS techniques to map coverage of roads in 2016 and was improved with data obtained from OpenStreetMaps and Google Map Maker (7, 25). The data was cleaned by removal of duplicates and correction of digitisation errors such as segments that fell short of connection points and sections that extended to water bodies. Roads were reclassified as primary, secondary, county, and rural roads (7, 25) (Supplementary Figure 2).

##### Land use

We considered different land covers that people have to navigate through where there are no roads due to variable levels of infrastructure development. Land cover at 10 × 10 m spatial resolution for 2020 produced by a deep learning model by European Space Agency Sentinel-2 imagery was used (45). It had eight land cover classes that covered Kenyan boundaries including water, trees, grass, flooded vegetation, crops, shrub, built-up area, and bare ground (43), as shown in Supplementary Figure 3.

##### Elevation and transport barriers

Slope of the land impedes walking and bicycling speeds when traveling. The slope was based on a digital elevation model sourced from the Shuttle Radar Topographic Mission at the 30 × 30 m spatial resolution available at the Regional Center for Mapping of Resources for Development (RCMRD) open data site (46). Finally, transport barriers that included lakes, major rivers, national parks, and reserves were considered impassable except where a road intersected a waterbody (7, 47) through a bridge, thus enabling access (Supplementary Figures 4, 5).

#### Population density

To estimate the population living within travel time thresholds relevant for policy, high spatial resolution population density maps are required. However, census data are usually available at coarse administrative units. To deal with the low spatial resolution data, dasymetric spatial modelling techniques have been developed (48) that redistribute census-based counts to high spatial resolution density maps informed by remotely sensed data such as land cover and night-time lights by shifting people from unlikely areas such as water bodies and assigning them to built-up areas. Such a population density map for 2019 (corresponding to Kenya's census year) was obtained from Worldpop's data portal (49).The population counts from Worldpop at 1 km spatial resolution were then extracted at county level using Zonal statistics in ArcMap version 10.5. The ratio between Kenya's 2019 census counts and the extracted counts from WorldPop were used to compute a scaling factor per county that was applied to the raster map to ensure that the totals match to 2019 Kenya's official census.The adjusted raster was then projected to 2021 using the 2009–2019 intercensal growth rate (32) at county level to obtain an estimate of population in 2021 (Supplementary Figure 1).

### Modelling travel time

We used a cost path distance algorithm (4) to compute travel time to the nearest health facility while accounting for the mode of transport, travel speeds, elevation, road classes and land cover for the entire country ignoring subnational boundaries. First, the land cover, protected areas, water bodies, rivers, and roads were spatially overlayed and merged through the “*merge land cover module*” of AccesMod (version 5.6.0) to create a friction raster surface. The friction surface was used with the location of health facilities and travel speeds to compute cumulative travel time from every populated location in Kenya based on WorldPop's population distribution maps toward (anisotropic) the closest health facilities *via* the shortest path.

Due to a lack of observational data on healthcare-seeking behaviour (19) to guide the parameterisation of our models, we relied on previous studies in similar contexts. Further, due to the complexity of modelling several modes of transport on a single road class, we only considered one mode of transportation per road class. However, we considered several modes of transport within a single journey between the household and the nearest health facility. These included walking, bicycling and motorised (motorbike and vehicles, both public and private) transport. We assumed that one walks to the nearest health facility without access to motorable roads or through motorised transport if a motorable road is adjacent to a residence and connects to a health facility. Otherwise, a person walks to the nearest bus stop to take a vehicle to the health care facility. The same concept applies to the bicycling mode of transport offered in the public sector (7).

Motorised transport was assigned to higher class roads (primary and secondary), cycling to county roads. In contrast, walking was assigned to rural roads and all other areas without roads were represented by different land cover classes (Table 1). The speeds used in each road class and land cover are defined in Table 1 and were based on previous studies (7, 25). The bicycling and walking speeds (Table 1) were further adjusted in AccesMod (version 5.6.0) to account for variation in velocity due to changing slope derived from the DEM. The walking speeds were corrected according to Tobler's formulation, an exponential function that describes how human walking speed varies with slope (50). On the other hand, bicycling power correction, which assumes increased speed due to negative slope does not exceed twice the speed on flat surfaces (51) was applied for bicycling speeds4F^4^.

**Table 1:** Travel modes and speeds adapted from previous studies (7, 47) that were used to compute travel time to the nearest health facility for each road type and land cover category.

**Land cover category**	**Speed km/h)**	**Transport mode**
Trees cover areas	2.5	Walking
Shrubs cover areas/sparse vegetation	4.5	Walking
Grassland	3.5	Walking
Cropland	4	Walking
Vegetation aquatic or regularly flooded	0	Walking
Bare areas and built-up areas	5	Walking
Open water	0	Walking
Primary roads	50	Motorized
Secondary roads	30	Motorized
County roads	10	Cycling
Rural roads	5	Walking

To explore the contribution of each sector toward healthcare accessibility in Kenya, three spatial access models were invoked, travel time to (i) private health facilities only, (ii) public health facilities only (iii) all health facilities (both public and private). The analyses were carried out at 300 × 300 m spatial resolution in AccesMod (version 5.6.0) which implements the cost path distance algorithm in its “accessibility module” (50) (Supplementary Figure 6).

### Sub-national policy-relevant metrics

We resolved the mean travel time and the proportion of the population within 1- and 2-h travel time of the nearest health facility by county and sub-county boundary. These are actionable units used for planning, resource allocation and benchmarking since the devolution of healthcare functions to county governments in Kenya. The administrative boundaries were updated from existing boundaries (52) based on the 2018–2022 County integrated development plans resulting in 298 sub-counties.

Disease tracers were chosen based on data availability, temporal concordance, spatial resolution, and among the leading causes of mortality among children under 5 years in Kenya (53). We then identified areas with a high prevalence of disease that were outside 1-h travel time to support prioritisation and targeting (54). These included (i) malaria prevalence among children aged 2–10 years in 2020 (55), (ii) lower respiratory infection(LRI) among under-fives in 2017 (56), and (iii) HIV prevalence among adults aged 15–49 years in 2017 (57). As an indicator of the overall population health, we also overlaid the travel time with the under-five mortality rate (U5MR) in 2015 (58). The disease prevalence tracers (55–57) and U5MR (58)were downloaded as gridded surfaces at 5 × 5 km spatial resolution. They had been interpolated through model-based geostatistical approaches applied to geolocated household survey data and relevant covariates.

## Results

Nationwide, a total of 13,912 facilities were identified from the DHIS2 and 13,238 facilities from the MHFL in 2021. Duplicate facilities (261), those indicated as closed in the MHFL (91) or facilities not offering routine clinical diagnosis and curative services (1,305) were identified and removed (Supplementary Table 1). The final database contained 13,579 facilities, 11,286 (83.1%) were reported on both the MHFL and DHIS2, 922 (6.8%) on the MHFL only and 1,371 (10.1%) on the DHIS2 platform only. Following detailed geocoding, only 611 (4.5%) facilities available on the combined DHIS2/MHFL database could not be geocoded, the majority (501, 82.0%) represented by the private sector (Table 2).

**Table 2:** Geo-coding of health facilities offering curative and preventative services to the general population.

	**Total facilities**	**Unable to geo-code**	**GPS**	**Google earth**
Ministry of health	6,071	83 (1.5%)	2,371 (39.1%)	3,617 (59.6%)
FBO	1184	15 (1.3%)	438 (37.0%)	731 (61.7%)
NGO	273	12 (4.4%)	36 (13.2%)	225 (82.4%)
Private sector	6051	501 (8.3%)	229 (3.8%)	5,321 (87.9%)
Total	13,579	611 (4.5%)	3,074 (22.6%)	9,894 (72.9%)

**Figure 1:**
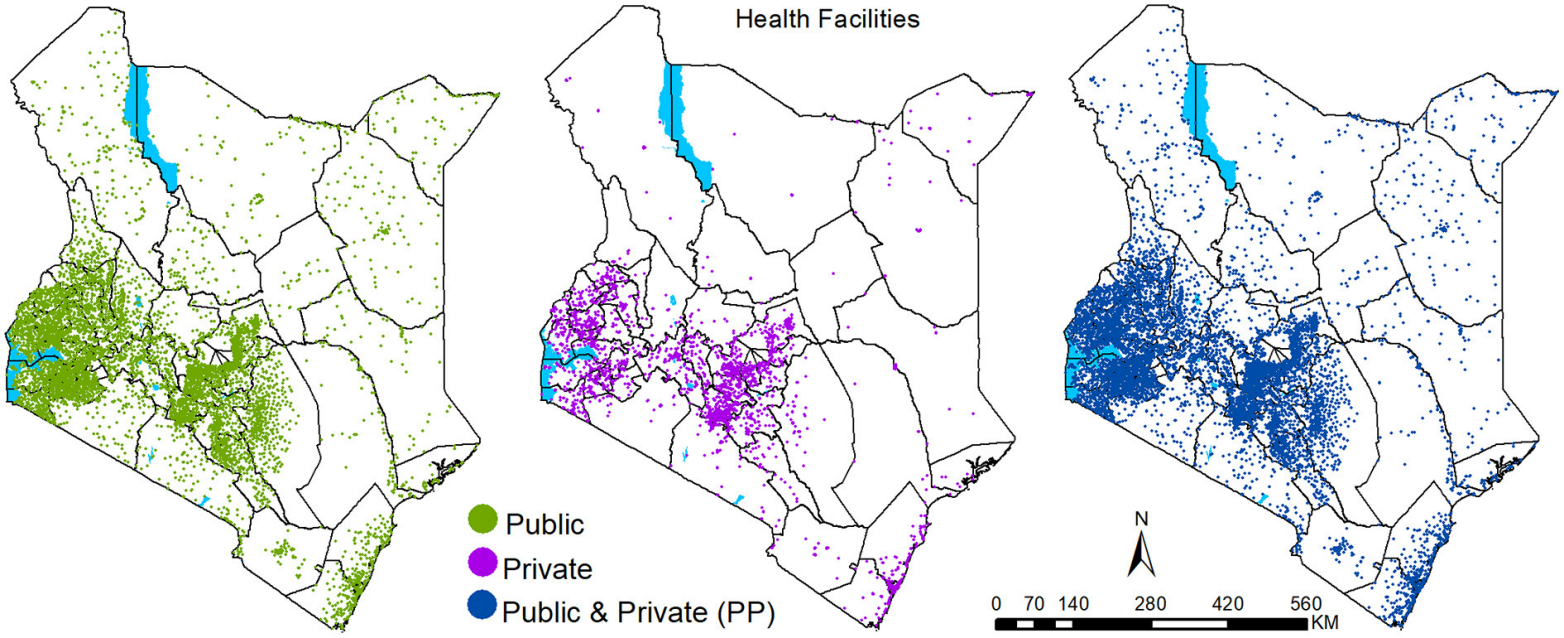
Health facilities' spatial distribution in 2021 by sector: Public (*n* = 7,418), Private (*n* = 5,550) and total (both public and private) health facilities (*n* = 12,968).

The 12,968 facilities that were geo-coded (Figure 1) are managed by MOH (5,988), NGO and FBO (1,430) and private-for-profit providers (5,550) and were linked to their county and sub-county locations and designated as either located in an urban or non-urban setting. Kitui (379), Nakuru (275), Makueni (265), Nairobi (267) and Meru (260) counties had the highest number of public health facilities. Among for-profit-private facilities, a majority (71%) were in 16 out of the 47 Kenyan counties. Nairobi (738), Kiambu (500), Nakuru (315) and Mombasa (297) had the highest numbers of private facilities, with Nyeri, Meru, Machakos and Kajiado having at least 200 health facilities each. Fifteen counties each had <50 private facilities, and among these, Lamu, Isiolo, West Pokot, Tana River and Elgeyo-Marakwet counties each had <20 private health facilities (Figure 1).

**Figure 2:**
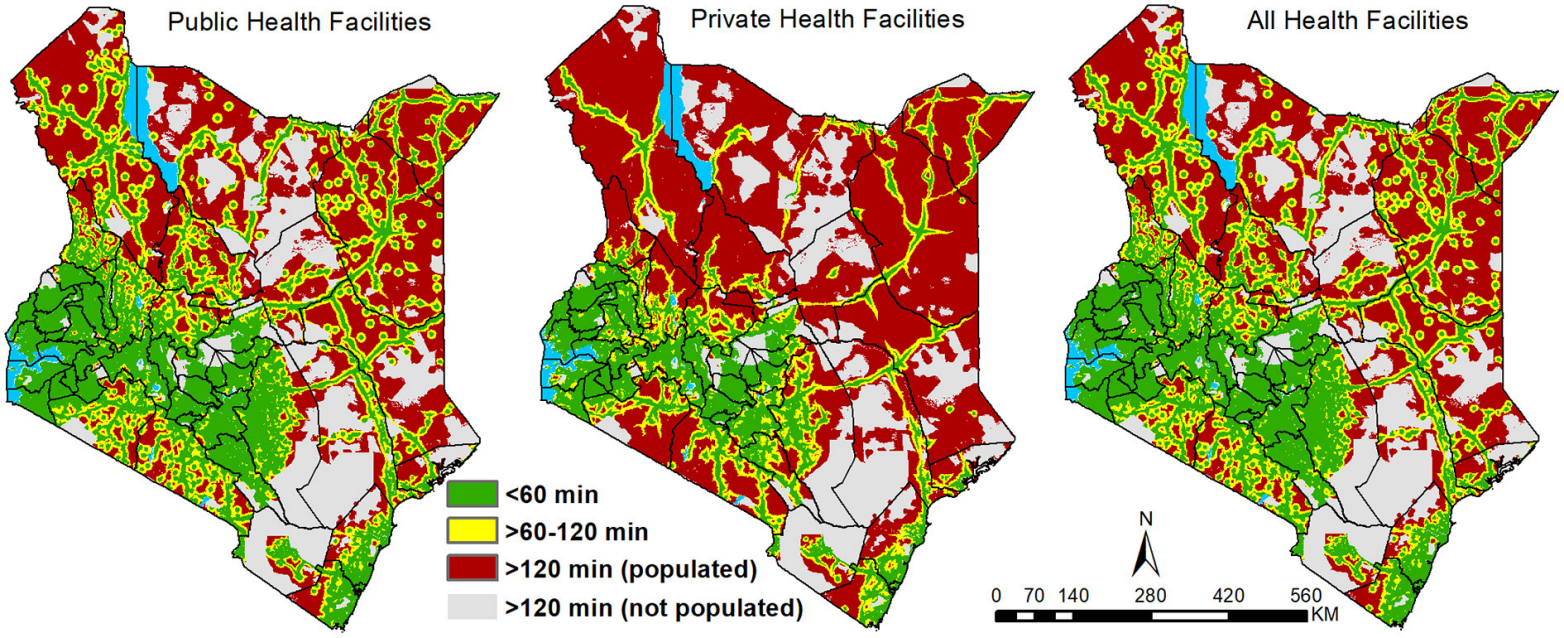
Travel time to the nearest public, private and combined (public and private) health facilities binned based on travel time and population density in Kenya in 2021.

At the national level, on average, public health facilities could be accessed within 130 min, within populated areas (Figure 2; Table 3). Private health facilities were comparably less accessible, with a national average of 254 min, due to their skewed presence in urban centers, especially in the 16 counties that account for 71% of the private facilities (Figure 2; Table 2). When the public and private sectors were combined, access improved marginally from an average of 130–128 min. Approximately 89.4 and 80.5% of the Kenya's 2021 population were within 1 h of the nearest public and private health facilities, respectively. When combined (private and public), the proportion within the same threshold was 89.6%. Only 6% were outside 2 h (marginalised) in each model except the private sector where 11% were marginalised (Table 2).

**Table 3:** The average travel time and proportion of population accessing care within 1 h of the nearest facility and those marginalized (outside 2 h) of public, private and all health facilities in Kenya at national level.

**Model**	**Average travel time (min-max) in minutes**	**Population 2021**		
		**Total**	**Within 1 h (%)**	**Outside 2 h (%)**
All heath facilities	128 (0–1,563)	48,948,358	43,833,250 (89.6)	2,731,882 (5.5)
Public health facilities	130 (0–1,563)		43,734,965 (89.4)	2,774,541 (5.6)
Private health facilities	254 (0–1,717)		39,402,625 (80.5)	5,280,839 (10.7)

At county level, travel time to the nearest public health facility was highly variable. In 62% of the counties, the average travel time to the nearest public service provider was <1 h while three counties (Turkana, Marsabit, Garissa) in Northern Kenya had an average of over 3 h. On average, public health facilities could be accessed in less than quarter of an hour in five counties of central (Nairobi, Kirinyaga) and western Kenya (Vihiga, Kisii, Nyamira) (Figure 2).

**Figure 3:**
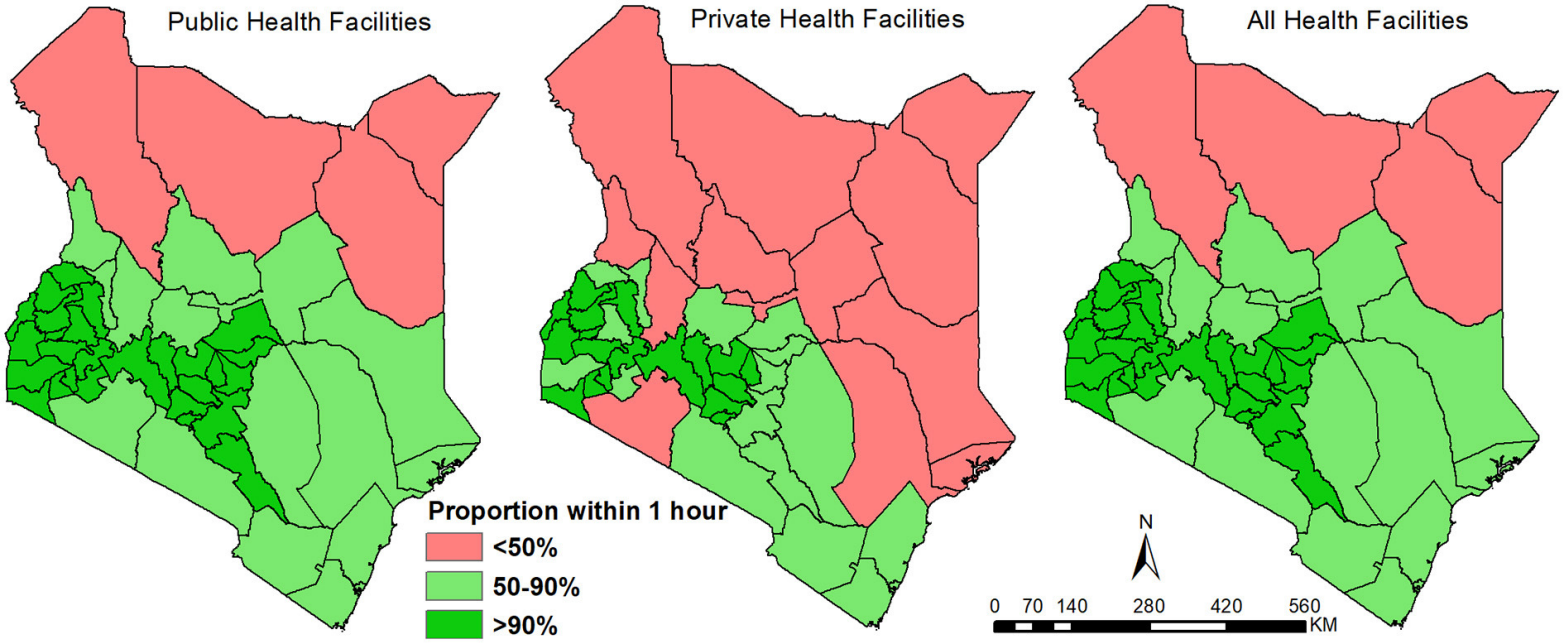
Proportion of population within a 1-h travel time of a public, private, and combined (public and private) in 2021 disaggregated by county.

In the public sector, accounting for population distribution, 27 counties (57%) have over 90% of their population within 1-h travel time of a public health facility. Bomet, Murang'a, Nyamira, Kiambu, Kisii and Nairobi counties had 99% of their population within the 1-h threshold (Figure 3). Most of the counties (43 counties) had at least 50% of their population within a 1-h threshold (Figure 3). The four counties with <50% of within 1-h included Turkana (37.8%), Marsabit (41.2%), Mandera (40.3%) and Wajir (38.2 %) (Figure 3). The most marginalised counties with at most 1 in 5 people (20%) outside 2 h travel catchment of a public health facility were Garissa, Isiolo, Lamu, Mandera, Marsabit, Samburu, Tana River, Turkana, and Wajir counties all located in northern or southeast regions (Supplementary Figure 7).

When considering access to private facilities, on average at the county level, only Nairobi and Vihiga counties had access within 15 min (Figure 2). Twenty-four additional counties (51%) had an average travel time to a facility of less than an hour. Kirinyaga, Nyamira, Mombasa, Kakamega, Kisii, Kiambu and Murang'a at <30 min on average while 10 counties had average access of over 3 h. After overlaying with population, 18 counties had over 90% of the population within an hour of a facility; Nyamira and Nairobi top the list at over 99.5%. Expanding the selection to counties with at least 50% of its population within an hour brings the count to 35 (74%) counties out of 47. Marginalised counties included Isiolo, Mandera, Marsabit, Samburu, Turkana, Wajir and West Pokot with more than half of its population living beyond 2 h travel time (Supplementary Figure 7).

When public and private facilities were combined, subnationally, improvement in access was not pronounced relative to public facilities only. Inclusion of private facilities improved access by reducing the average travel time to the nearest facility by at least a minute in 39 of the 47 counties. Tana River, Garissa, Lamu, Taita Taveta, Kwale, Laikipia and Kilifi had at least 4 min reductions. The number of counties whose average travel time was ≤15 min travel time increased from 5 to 8 counties with the addition of Murang'a, Kakamega and Mombasa. However, the counties with an average of 3 h remained unchanged despite a slight reduction in the mean travel time in each of these counties. Similarly, the number of counties with over 90% (27 counties), 50% (43 counties) or below 50% (4 counties) of their population within 1-h travel time remained unchanged (Figure 3), with the same going for the most marginalised counties (Supplementary Figure 7; Supplementary Tables 2, 3). Approximately 100% of the population residing in urban areas had access to a facility within 1 h which accounts for 13% of the total national population within 1 h of any health facility (Table 4). Additionally, when restricting comparison of average travel time to the 16 counties in which private facilities are primarily distributed, public facilities could be accessed within an average time of 53 min while private facilities had an average of 87 min.

**Table 4:** Distribution of all health facilities and summary of populations within 1 h of the nearest facility by urban and non-urban spatial extents.

**Area**	**Health facilities (%)**	**Population within 1 h (%) by area**	**%Population within 1 h nationally**
Urban	3,329 (26)	5,910,612 (99)	13
Non-Urban	9,639 (74)	37,922,638 (88)	86

**Figure 4:**
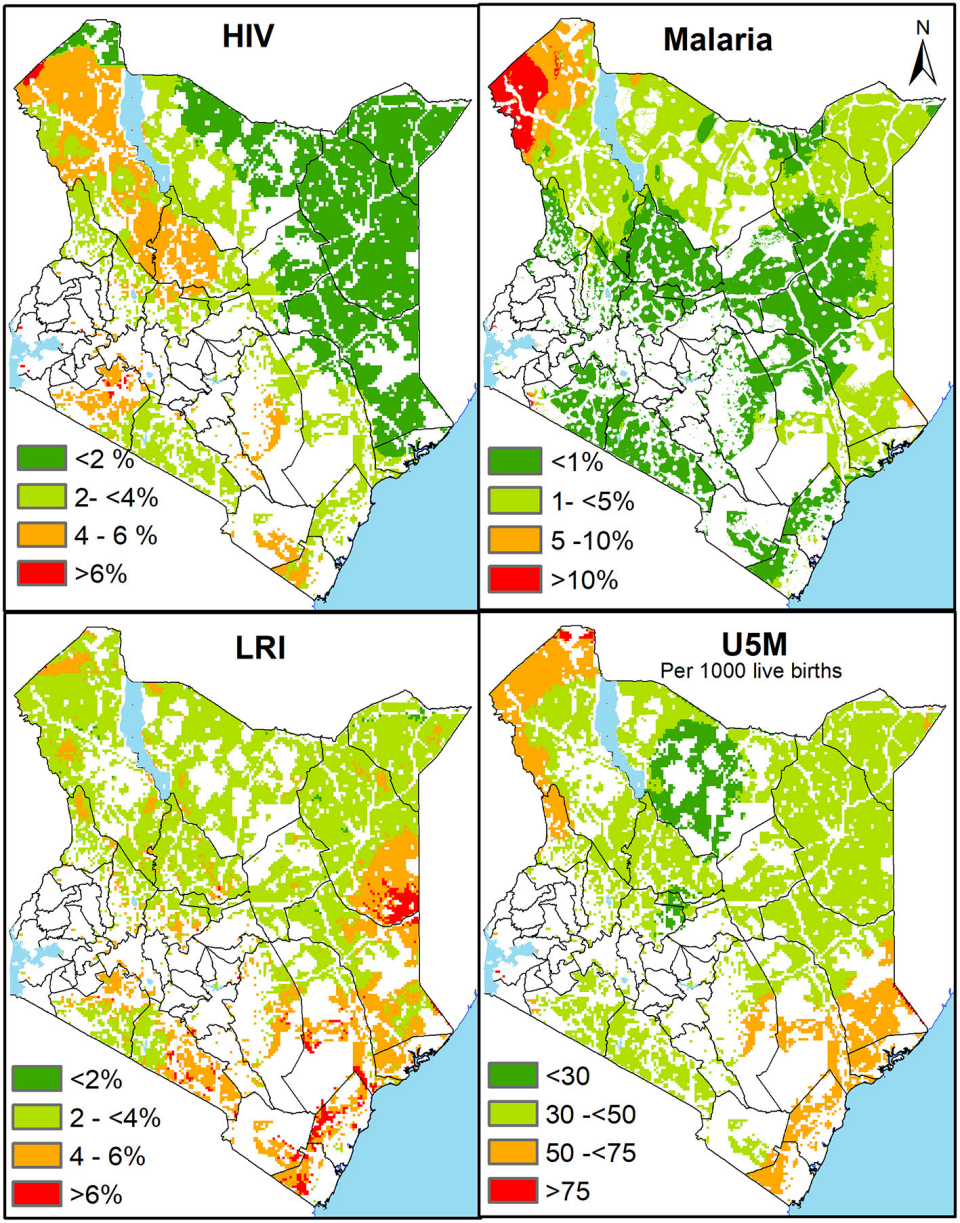
The spatial variation of infection prevalence of HIV, Malaria, LRI and under five mortality in areas outside 1-h catchment of the nearest health facility (public and private) in 2021. Red represents areas of dual burden with high disease prevalence or mortality and over 1-h distance from health facility while green shades represent regions of lower disease prevalence or mortality outside 1-h.

Several counties bore a dual burden (Figure 4). These counties had proportionately large swaths outside 1-h and at the same time with a high infection prevalence of HIV (Turkana, Samburu, Narok and partly Kwale), malaria (Turkana), LRIs (Kwale, Kilifi, Lamu, Garissa, Mandera, Kajiado, partly in Narok and Kitui) and under-fiver mortality rates (Turkana, Kwale, Kilifi, Lamu, Garissa, partly Tana River and West Pokot). What is clear is that some counties such as Turkana have higher disease prevalence for several diseases, yet the majority live more than 1-h of the nearest public health facility.

## Discussion

An understanding of spatial distribution of health facilities by sector has implications on the service provision, quality of care and economic considerations at the household level in accessing care. Continuous updating and geocoding of health facilities allows for an improved understanding of service demand and quantifying subnational disparities in access to health care to identify gaps at a micro level. Here for the first time, we improved the completeness of Kenya's MHFL and fidelity of the geographic location of both public and private health facilities offering curative or preventative health services to the general population while characterising disparities in geographic access.

The importance of an updated facility database cannot be over emphasised. Emerging infectious disease outbreaks, including the SARS-CoV-2 pandemic and Ebola, served as a stark reminder of the significance, and inadequacies, of real-time spatially linked data needs to track infectious diseases in SSA (60, 61). The COVID pandemic demonstrated the benefit of a robust geocoded health facility data with real-time information on health services and capacity in health facilities. For example, critical services such as the availability of oxygen and the number of high dependency and intensive care bed capacities, is a major concern during the ongoing COVID-19 pandemic. This further highlights the need to independently assess, maintain and update information on services offered by health facilities for a real time and complete picture of the health system's capacity. Further, sample surveys of health facilities (62–65) require a complete, updated census of both public and private service providers as part of sub-nationally weighted sampling frames. The analysis of routine data at sub-national administrative levels, or finer resolution facility levels, necessitates understanding the universe of public and private health service providers and accuracy in the location information attributed to each facility.

The intra-operability between MHFL and routine health data (DHIS2) is fundamental to the reliability of sub-national service and disease reporting. Since 2006, the roll-out of DHIS2 across SSA has substantively changed the capacity to link routine disease reporting and commodity supply data in space and time (66, 67). There is increasing use of sub-national, fine resolution disease and service delivery reporting in Kenya, ranging from coverage estimation of maternal and child health indicators (36), EPI access (7), malaria test-positivity (68), the impact of COVID 19 on service delivery (69, 70) all which require an accurate MHFL. The inconsistencies and mismatches between the number of health facilities in MHFL and DHIS2 should give an impetus to hasten the harmonisation of these two platforms, given their role in national public health.

Increasing use of spatial data at sub-national levels of the health system responsible for updating service provider listings will inevitably lead to improved spatial data quality, as with usability of DHIS2 data more broadly. Through a labour-intensive exercise, we could geo-code 95.5% of the combined MHFL/DHIS2 health facilities, providing an updated spatial distribution of facilities in Kenya. County-level training would improve the use of GPS, smart-phone technology to confirm the locations of service providers and build the capacity and use of GIS at sub-national levels.

We have demonstrated the value of the assembled database to provide, for the first time, spatial accessibility metrics to both public and private facilities in Kenya while highlighting areas with a dual burden of poor access and high disease burden. Improved healthcare utilisation for routine and emergency services is a function of good geographical access between households and service provision sites and requires regular interrogations to identify and target areas that have been left behind. This is essential and in line with The Kenya Health Sector Strategic Investment Plan, which prioritises identifying health infrastructure gaps while ensuring health services are located physically near the population residences at a recommended distance of 5 km (1 h) comparable to recommended international thresholds set by the WHO (7, 49, 54, 71).

Nationally, in 2021, geographic access is substantially high, with nine out of ten people living within 1-h travel time of any nearest health facility or a public health facility. Thus, private health facilities did not seem to change overall geographic accessibility at the national level. This could be due to several reasons. Of note, many private facilities are located near public service providers (Figure 1). For example, at least a fifth of the private facilities are within 500 meters of a public facility. This is further compounded by the fact that most private facilities are located within urban areas with a wider and improved road network, better access to motorised transport, higher speeds relative to rural areas, contributing to the seemingly low impact of the private sector in improving geographic access. Notably, despite a county having many private health facilities, the facilities are concentrated within the main urban area (central business district), thus resulting in overlaps in the population served by the public. This distribution might be due to a lack of incentive for the private sector to serve the rural populations or perhaps limited capacity to expand across the country except for a few large entities that also leverage on established reputation.

The national level statistics are important for national level policy, advocacy, and regional comparison. Despite their importance, national statistics mask subnational heterogeneity, popularly known as *masking the unfinished health agenda* (72). For example, the proportion within 1 h was 90% at the national level, however, the spatial variation across the counties ranged between 38 and 100% when considering both public and private facilities (Figure 3). The subnational distribution is similar to the historical patterns of healthcare utilisation, malnutrition indicators, poverty, access to water and sanitation that were observed during the millennium development period (73). Counties located in central and western Kenya have the highest proportion of the population that can access any health facility within 1-h and is likely associated with greater historic investment, high levels of urbanisation and high population density (74).

Approximately 2.7 million people (6%) are marginalised (living outside 2 h) from routine care offered by both the public and private sector. The counties with majority of the marginalised population are in northern parts of Kenya and include Garissa, Isiolo, Lamu, Mandera, Marsabit, Turkana and Wajir. These counties can be characterised as arid and semi-arid areas (ASALs) with low population density and predominantly occupied by communities that practice nomadic pastoralism. Periodic upsurges of security concerns have further hindered economic growth and development in the region. The marginalisation from basic health services in these areas is historic (7, 27, 74) and remains evident in the present day despite some investments in this region. Continued investment in infrastructure development, other forms of health provision such as mobile clinics (or makeshift clinics such as schools, churches, mosques) by the government and regular medical camps by non-governmental organisations will be needed to bridge the gap in accessibility. Furthermore, investigations on the unique nature of healthcare provision in ASAL settings will improve our understanding of the unique dynamics at play to inform better healthcare provision for this region.

The private sector continues to grow in Kenya, with increasing franchised, private consortia offering services across the country including the Aga Khan health care5F^5^, African Air Rescue6F^6^, Bliss Health Care Group7F^7^, Equity Afya8F^8^, the Tudor Health Care group9F^9^ and a proliferation of private practitioners. However, despite its inclusion for the first time in accessibility models in Kenya, its overall influence on physical access was little. Nevertheless, its contribution goes beyond the geographical accessibility agenda. The private-for-profit sector plays an important role in service provision (30, 75). They offer greater flexibility in location especially in urban areas an important contribution given the high rates of urbanisation being seen in SSA and particularly in Kenya. The private sector provides a wider selection of service provision and greater efficiency in providing care, albeit at a higher cost compared to public facilities. In Kenya, it has been shown that 47% of households in the poorest quantile of Kenyans were reported to use a private facility when a child is sick in 2005 (76) and increasing utilisation of private facilities among the poor (30, 59). The sector's increasing market share underscores its possible contribution in increasing access to healthcare, supplementing care and services offered by the public health sector and in ensuring the achievement of UHC and SDGs (77, 78). Acknowledging the challenges in public facilities, the private sector offers an alternative route and choice to seek care. Private facilities can be used as means to decongest the most overcrowded public facilities when the government provides specific commodities free of charge to all private facilities and by contracting them to provide certain types of care (78–80). With such structures in place, further incentives to extend their services to rural and under-served regions would improve the access and quality of services available to communities that have been left behind.

### Strengths and limitations

This study has several strengths, the first of which is in the assembly, harmonisation and geocoding of a comprehensive health facility list of all operational public and private health facilities in 2021. Secondly, it is the first study to assess geographic access to both public and private health facilities in Kenya. Further, this study takes into account the barriers of travel (land use, rivers, protected areas), modes of transport and travel speeds that influence access to a facility and offers more realistic access estimates compared to straight-line distances. Lastly, this study highlights populations that experience a dual burden of disease and marginalization (outside 1 h) for targeting.

On the other had, this study did not cover other dimensions of access to health care such as affordability, acceptability, type, and quality of services. We did not consider specific healthcare provision sites, including community outreach programmes and mobile clinics, which may have provided greater access to health services in remote areas. We excluded 4.5% of facilities that could not be geocoded, which may have affected the proximity metrics in some counties. Given the dynamic nature of health facility operations (closures and openings) especially the private health facilities, sourcing health facility data exclusively from DHIS2 and MHFL may have led to the omission of some facilities that were not part of these two listings. We extracted speeds from similar studies and did not account for care-seeking behaviour. Due to the lack oflocation of origin (households/villages) of patients we assumed that the nearest facility was used despite the well-known bypassing mechanisms influenced by factors such as cost or perceived quality of care which may result in our model overestimating access. The dataset to classify facilities and populations in urban areas does not capture the urban-rural continuum thus classification was limited to either urban or non-urban. Finally, we did not account for disruptions linked to the COVID-19 pandemic. However, the pandemic increased pressure on the already constrained health system.

The use of a cost distance algorithm is a pragmatic choice in national-level studies, given the limited care-seeking data at a large scale. However, further studies collating data on where the need to seek was triggered and where it was sought, the mode(s) of transport that were used, travel speeds, time of the day, weather conditions, traffic conditions, reasons for seeking care, waiting time at the facility,competition between facilities and related information such as security are necessary (19, 81). These data would strengthen further investigations to improve access in the hotspots highlighted the national study and other access studies tied to particular health outcomes and services. This will allow improved and localised policies for targeting gaps during care seeking (81). Additional investments are needed to assemble and geolocate all private health facilities to build on the current existing database of public health facilities in SSA (20). Further, attribute information attached to each facility (both public and private) will increase their utility in assessing access to care for specific services with growing demand, such as cancer treatment facilities and better defining health facility service catchments.

## Conclusions

For the first time, a comprehensive inventory of both public and private health facilities was assembled and geocoded. Using this database, we show subnational heterogeneities in geographic access and colocation of areas outside 1-h of a health facility and regions with high disease prevalence. The database will be helpful for generating subnational metrics based on routine data. At the same time, the geographically marginalised areas should be prioritised in resource allocation by the subnational governments toward reducing health inequities and pathways to attaining UHC.

## Data availability statement

The data analyzed in this study is subject to the following licenses/restrictions: Aggregated DHIS2 data is available online with access provided by Kenya Ministry of Health. The national master health facility lists is provided through the MHFL website http://kmhfl.health.go.ke/#/home. The datasets used and/or analysed during the current study are available to others from these sources on reasonable request. Requests to access these datasets should be directed to Ministry of Health KHIS https://hiskenya.org/dhis-web-commons/security/login.action and KMHFL, kmhfl@health.go.ke.

## Author contributions

AKM: data curation, geo-coding, formal analysis, investigation, methodology, software, validation, visualisation, writing–original draft, and writing–review and editing. LS: data curation, geo-coding, and writing–review and editing. EM: data curation, software, and writing–review and editing. RWS and EAO: conceptualisation, data curation, funding acquisition, resources, validation, and writing–review and editing. PMM: conceptualisation, funding acquisition, investigation, methodology, project administration, supervision, validation, visualization, writing–original draft, and writing–review and editing. All authors contributed to the article and approved the submitted version.

## Funding

PMM is supported by the Royal Society Newton International Fellowship (NIF/R1/201418). LS was funded under a grant to Dr. Victor Alegana by the Wellcome Trust (Number 211208). EAO is supported as a Wellcome Trust Intermediate (Number 201866) and Senior Fellow (Number 224272). RWS is supported as a Wellcome Trust Principal Fellow (Number 212176) that also provided support for AKM, LS, and EM. All authors are grateful to the support of the Wellcome Trust to the Kenya Major Overseas Programme (Number 203077).

## Conflict of interest

The authors declare that the research was conducted in the absence of any commercial or financial relationships that could be construed as a potential conflict of interest.

## Publisher's note

All claims expressed in this article are solely those of the authors and do not necessarily represent those of their affiliated organizations, or those of the publisher, the editors and the reviewers. Any product that may be evaluated in this article, or claim that may be made by its manufacturer, is not guaranteed or endorsed by the publisher.

## Footnotes

^1^https://tasks.hotosm.org/ (accessed September 24, 2021).

^2^https://www.missingmaps.org/ (accessed September 24, 2021).

^3^https://www.youthmappers.org/ (accessed September 24, 2021).

^4^http://bikecalculator.com/ (accessed November 14, 2021).

^5^https://www.akdn.org (accessed September 24, 2021).

^6^https://aar-healthcare.com/ (accessed September 24, 2021).

^7^https://www.blissmedicalcentre.com/ (accessed September 24, 2021).

^8^https://www.equityafia.co.ke/ (accessed September 24, 2021).

^9^https://www.tudorhealthcare.com/ (accessed September 24, 2021).

## Abbreviations

DHIS2: district health information systems version 2
DEM: digital elevation model
EPI: expanded programme of immunization
ESA: European space agency
FBO: faith-based organization
GPS: global positioning system
HOT: humanitarian openstreetmap team
KWTRP: KEMRI-wellcome trust research programme
MHFL: master health facility list
MOH: ministry of health
NASA: national aeronautics and space administration
NGO: non-governmental organization
RCMRD: regional centre for mapping of resources for development
SDG: sustainable development goals
SSA: sub-saharan Africa
UHC: universal health coverage
WHO: world health organization.

## References

[1] Evans DB Hsu J Boerma T. Universal health coverage and universal access. Bull World Health Organ. (2013) 91:546. 10.2471/BLT.13.125450 23940398 PMC3738317

[2] Mwangi C. Accessibility to the Kenyan Health Care System: Barriers to Accessing Proper Health Care [Dissertation]. Helsinki: Arcada University of Applied Sciences (2014).

[3] Bakibinga P Kisia L Atela M Kibe PM Kabaria C Kisiangani I. Demand and supply-side barriers and opportunities to enhance access to healthcare for urban poor populations in kenya: a qualitative study. BMJ Open. (2022) 12:e057484. 10.1136/bmjopen-2021-057484 35523490 PMC9083429

[4] Ouma P Macharia PM Okiro E Alegana V. Methods of measuring spatial accessibility to health care in uganda. Pract Health Geography. (2021) 1:77–90. 10.1007/978-3-030-63471-1_6

[5] Higgs G. A literature review of the use of gis-based measures of access to health care services. Health Serv Outcomes Res Methodol. (2004) 5:119–39. 10.1007/s10742-005-4304-729772871

[6] Penchansky R Thomas JW. The concept of access: definition and relationship to consumer satisfaction. Med Care. (1981) 1981:127–40. 10.1097/00005650-198102000-00001 7206846

[7] Joseph NK Macharia PM Ouma PO Mumo J. Jalang'o R, Wagacha PW, et al. Spatial access inequities and childhood immunisation uptake in Kenya. BMC Public Health. (2020) 20:1407. 10.1186/s12889-020-09486-8 32933501 PMC7493983

[8] Kadobera D Sartorius B Masanja H Mathew A Waiswa P. The effect of distance to formal health facility on childhood mortality in rural tanzania, 2005–2007. Glob Health Action. (2012) 5:19099. 10.3402/gha.v5i0.19099 23151364 PMC3495250

[9] Karra M Fink G Canning D. Facility distance and child mortality: a multi-country study of health facility access, service utilization, and child health outcomes. Int J Epidemiol. (2017) 46:817–26. 10.1093/ije/dyw062 27185809

[10] McKinnon B Harper S Kaufman JS Abdullah M. Distance to emergency obstetric services and early neonatal mortality in e thiopia. Trop Med Int Health. (2014) 19:780–90. 10.1111/tmi.12323 24750556

[11] Juran S Broer PN Klug SJ Snow RC Okiro EA Ouma PO. Geospatial mapping of access to timely essential surgery in sub-saharan Africa. BMJ Global Health. (2018) 3:e000875. 10.1136/bmjgh-2018-000875 30147944 PMC6104751

[12] Ouma PO Maina J Thuranira PN Macharia PM Alegana VA English M. Access to emergency hospital care provided by the public sector in sub-saharan Africa in 2015: a geocoded inventory and spatial analysis. Lancet Global Health. (2018) 6:e342–50. 10.1016/S2214-109X(17)30488-6 29396220 PMC5809715

[13] Kim ET Singh K Speizer IS Angeles G Weiss W. Availability of health facilities and utilization of maternal and newborn postnatal care in rural malawi. BMC Pregnancy Childbirth. (2019) 19:503. 10.1186/s12884-019-2534-x 31847872 PMC6918704

[14] Wong KL Brady OJ Campbell OM Banke-Thomas A Benova L. Too poor or too far? Partitioning the variability of hospital-based childbirth by poverty and travel time in kenya, malawi, nigeria and tanzania. Int J Equity Health. (2020) 19:15. 10.1186/s12939-020-1123-y 31992319 PMC6988213

[15] Wong KL Benova L Campbell OM. A Look back on how far to walk: systematic review and meta-analysis of physical access to skilled care for childbirth in sub-saharan Africa. PLoS ONE. (2017) 12:e0184432. 10.1371/journal.pone.0184432 28910302 PMC5598961

[16] Tegegne TK Chojenta C Loxton D Smith R Kibret KT. The impact of geographic access on institutional delivery care use in low and middle-income countries: systematic review and meta-analysis. PLoS ONE. (2018) 13:e0203130. 10.1371/journal.pone.0203130 30161201 PMC6117044

[17] WHO. Creating a Master Health Facility List. Geneva: World Heal Organ (2013). p. 1–49.

[18] Nilsen K Tejedor-Garavito N Leasure DR Utazi CE Ruktanonchai CW Wigley AS. A review of geospatial methods for population estimation and their use in constructing reproductive, maternal, newborn, child and adolescent health service indicators. BMC Health Serv Res. (2021) 21:370. 10.1186/s12913-021-06370-y 34511089 PMC8436450

[19] Macharia PM Ray N Giorgi E Okiro EA Snow RW. Defining service catchment areas in low-resource settings. BMJ Global Health. (2021) 6:e006381. 10.1136/bmjgh-2021-006381 34301676 PMC8728360

[20] Maina J Ouma PO Macharia PM Alegana VA Mitto B Fall IS. A spatial database of health facilities managed by the public health sector in sub saharan Africa. Sci Data. (2019) 6:1–8. 10.1038/s41597-019-0142-2 31346183 PMC6658526

[21] South A Dicko A Herringer M Macharia PM Maina J Okiro EA. A reproducible picture of open access health facility data in Africa and R tools to support improvement. Wellcome Open Res. (2020) 5:157. 10.12688/wellcomeopenres.16075.1 33437875 PMC7780339

[22] Geldsetzer P Reinmuth M Ouma PO Lautenbach S Okiro EA Bärnighausen T. Mapping physical access to health care for older adults in sub-saharan africa and implications for the COVID-19 response: a cross-sectional analysis. Lancet Healthy Longevity. (2020) 1:e32–42. 10.1016/S2666-7568(20)30010-6 34173615 PMC7574846

[23] Alegana VA Maina J Ouma PO Macharia PM Wright J Atkinson PM. National and sub-national variation in patterns of febrile case management in sub-saharan Africa. Nat Commun. (2018) 9:4994. 10.1038/s41467-018-07536-9 30478314 PMC6255762

[24] Ocholla IA Agutu NO Ouma PO Gatungu D Makokha FO Gitaka J. Geographical accessibility in assessing bypassing behaviour for inpatient neonatal care, bungoma county-kenya. BMC Pregnancy Childbirth. (2020) 20:287. 10.1186/s12884-020-02977-x 32397969 PMC7216545

[25] Macharia PM Odera PA Snow RW Noor AM. Spatial models for the rational allocation of routinely distributed bed nets to public health facilities in western kenya. Malar J. (2017) 16:367. 10.1186/s12936-017-2009-3 28899379 PMC5596856

[26] Noor A Gikandi P Hay S Muga R Snow R. Creating spatially defined databases for equitable health service planning in low-income countries: the example of kenya. Acta Trop. (2004) 91:239–51. 10.1016/j.actatropica.2004.05.003 15246930 PMC2673552

[27] Noor AM Alegana VA Gething PW Snow RW. A spatial national health facility database for public health sector planning in kenya in 2008. Int J Health Geogr. (2009) 8:13. 10.1186/1476-072X-8-13 19267903 PMC2666649

[28] Ouma PO Agutu NO Snow RW Noor AM. Univariate and multivariate spatial models of health facility utilisation for childhood fevers in an area on the coast of kenya. Int J Health Geogr. (2017) 16:34. 10.1186/s12942-017-0107-7 28923070 PMC5604359

[29] Maina JK Macharia PM Ouma PO Snow RW Okiro EA. Coverage of routine reporting on malaria parasitological testing in kenya, 2015–2016. Glob Health Action. (2017) 10:1413266. 10.1080/16549716.2017.1413266 29261450 PMC5757226

[30] Barnes J O'Hanlon B Feeley III. F, McKeon K, Gitonga N, Decker C. Private Health Sector Assessment in Kenya. Washington, DC: World Bank Publications (2010).

[31] Kenya Go. The Constitution of Kenya. Nairobi: National Council for Law Reporting (2010).

[32] KNBS. Kenya Population and Housing Census Reports 2020. Nairobi: Kenya National Bureau of Statistics (2019).

[33] Health Mo. Kenya Health Sector Strategic and Investment Plan (Khssp). Nairobi: Ministry of Health (2018).

[34] Butler RJ. Atlas of Kenya: A Comprehensive Series of New and Authentic Maps Prepared from the National Survey and Other Governmental Sources with Gazetteer and Notes on Pronunciation & Spelling: Survey of Kenya. Nairobi: Survey of Kenya (1959).

[35] World Health Organization. HealthMap: WHO/UNICEF Joint Programme on Data Management and Mapping for Public Health. World Health Organization (1999). Available online at: https://apps.who.int/iris/handle/10665/66438 (accessed October 24, 2022).

[36] Maina I Wanjala P Soti D Kipruto H Droti B Boerma T. Using health-facility data to assess subnational coverage of maternal and child health indicators, kenya. Bull World Health Organ. (2017) 95:683. 10.2471/BLT.17.194399 29147041 PMC5689197

[37] MHFL. Kenya Master Health Facility List. (2021). Available online at: http://kmhfl.health.go.ke/#/home (accessed August 18, 2021).

[38] Herfort B Lautenbach S de Albuquerque JP Anderson J Zipf A. The evolution of humanitarian mapping within the openstreetmap community. Sci Rep. (2021) 11:1–15. 10.1038/s41598-021-82404-z 33542423 PMC7862441

[39] Ahlers D. Assessment of the accuracy of geonames gazetteer data. Proceedings of the 7th Workshop on Geographic Information Retrieval. New York (2013).

[40] Acheson E De Sabbata S Purves RS. A quantitative analysis of global gazetteers: patterns of coverage for common feature types. Comput Environ Urban Syst. (2017) 64:309–20. 10.1016/j.compenvurbsys.2017.03.007

[41] ESA. Satellite Imagery Key to Powering Google Earth. Available online at: https://www.esa.int/Applications/Observing_the_Earth/Satellite_imagery_key_to_powering_Google_Earth (accessed August 24, 2021).

[42] DHIS2. Khis-Dhis2. (2021). Available online at: https://hiskenya.org/dhis-web-commons/security/login.action (accessed August 18, 2021).

[43] Karuri J Waiganjo P Daniel O Manya A. Dhis2: the tool to improve health data demand and use in kenya. J Health Inform Dev Count. (2014) 8:38–60.

[44] Macharia P, Mumo, E, Okiro, EA,. Kenya Urban Centres 2019. (2021). Available online at: https://figshare.com/articles/dataset/Kenya_Urban_Centres_2019/13308773 (accessed October 11, 2022).

[45] Karra K Kontgis C Statman-Weil Z Mazzariello JC Mathis M Brumby SP. Global land use/land cover with sentinel 2 and deep learning. 2021 IEEE International Geoscience and Remote Sensing Symposium IGARSS. Brussels (2021).

[46] RCMRD. Kenya Srtm Dem 30meters. Available online at: https://opendata.rcmrd.org/datasets/kenya-srtm-dem-30meters/explore (accessed November 14, 2021).

[47] Macharia PM Mumo E Okiro EA. Modelling geographical accessibility to urban centres in kenya in 2019. PLoS ONE. (2021) 16:e0251624. 10.1371/journal.pone.0251624 33989356 PMC8127925

[48] Stevens FR Gaughan AE Linard C Tatem AJ. Disaggregating census data for population mapping using random forests with remotely-sensed and ancillary data. PLoS ONE. (2015) 10:e0107042. 10.1371/journal.pone.0107042 25689585 PMC4331277

[49] Worldpop. (2021). Available online at: https://www.worldpop.org/ (accessed November 2, 2021).

[50] Ray N. Ebener S. Accessmod 30: computing geographic coverage and accessibility to health care services using anisotropic movement of patients. Int J Health Geog. (2008) 7:63. 10.1186/1476-072X-7-63 19087277 PMC2651127

[51] Zorn A,. Bicycle Velocity Power Calculator. (2008). Available online at: http://bikecalculator.com/ (accessed November 14, 2021).

[52] Macharia PJ, Noel K;, Okiro, Emelda,. Sub Counties of Kenya Based on County Intergrated Development Plans(Cidps). (2020). Available online at: 10.6084/m9.figshare.12501455.v1 (accessed March 15, 2021).

[53] Compare G Hub V. Institute for Health Metrics and Evaluation (Ihme). Seattle: University of Washington (2015).

[54] Health Mo. Kenya Health Sector Strategic and Investment Plan (Khssip). Nairobi: Ministry of Health (2014).

[55] Alegana VA Macharia PM Muchiri S Mumo E Oyugi E Kamau A. Plasmodium falciparum parasite prevalence in east africa: updating data for malaria stratification. PLOS Global Public Health. (2021) 1:e0000014. 10.1371/journal.pgph.0000014 35211700 PMC7612417

[56] Reiner RC Welgan CA Casey DC Troeger CE Baumann MM Nguyen QP. Identifying residual hotspots and mapping lower respiratory infection morbidity and mortality in African children from 2000 to 2017. Nat Microbiol. (2019) 4:2310–8. 10.1038/s41564-019-0562-y 31570869 PMC6877470

[57] Dwyer-Lindgren L Cork MA Sligar A Steuben KM Wilson KF Provost NR. Mapping hiv prevalence in sub-saharan Africa between 2000 and 2017. Nature. (2019) 570:189–93. 10.1038/s41586-019-1200-9 31092927 PMC6601349

[58] Golding N Burstein R Longbottom J Browne AJ Fullman N Osgood-Zimmerman A. Mapping under-5 and neonatal mortality in Africa, 2000–15: a baseline analysis for the sustainable development goals. Lancet. (2017) 390:2171–82. 10.1016/S0140-6736(17)31758-0 28958464 PMC5687451

[59] Wambiya EOA Otieno PO Mutua MK Donfouet HPP Mohamed SF. Patterns and predictors of private and public health care utilization among residents of an informal settlement in nairobi, kenya: a cross-sectional study. BMC Public Health. (2021) 21:850. 10.1186/s12889-021-10836-3 33941131 PMC8091493

[60] Lal A Ashworth HC Dada S Hoemeke L Tambo E. Optimizing pandemic preparedness and response through health information systems: lessons learned from ebola to COVID-19. Disaster Med Public Health Prep. (2022) 16:333–40. 10.1017/dmp.2020.361 33004102 PMC7642496

[61] Lal A Erondu NA Heymann DL Gitahi G Yates R. Fragmented health systems in COVID-19: rectifying the misalignment between global health security and universal health coverage. Lancet. (2021) 397:61–7. 10.1016/S0140-6736(20)32228-5 33275906 PMC7834479

[62] O'Neill K Takane M Sheffel A Abou-Zahr C Boerma T. Monitoring service delivery for universal health coverage: the service availability and readiness assessment. Bull World Health Organ. (2013) 91:923–31. 10.2471/BLT.12.116798 24347731 PMC3845262

[63] IHME. Health Service Provision in Kenya: Assessing Facility Capacity, Costs of Care, and Patient Perspectives. Seattle, WA: IHME (2014).

[64] Health Mo. Harmonised Health Facility Assessment. Nairobi: Ministry of Health (2019).

[65] Zurovac D Machini B Kiptui R Memusi D Amboko B Kigen S. Monitoring health systems readiness and inpatient malaria case-management at kenyan county hospitals. Malar J. (2018) 17:213. 10.1186/s12936-018-2364-8 29843717 PMC5975267

[66] Dehnavieh R Haghdoost A Khosravi A Hoseinabadi F Rahimi H Poursheikhali A. The district health information system (Dhis2): a literature review and meta-synthesis of its strengths and operational challenges based on the experiences of 11 countries. Health Inform Manag J. (2019) 48:62–75. 10.1177/1833358318777713 29898604

[67] Farnham A Utzinger J Kulinkina AV Winkler MS. Using district health information to monitor sustainable development. Bull World Health Organ. (2020) 98:69. 10.2471/BLT.19.239970 31902965 PMC6933431

[68] Alegana VA Suiyanka L Macharia PM Ikahu-Muchangi G Snow RW. Malaria micro-stratification using routine surveillance data in western kenya. Malar J. (2021) 20:22. 10.1186/s12936-020-03529-6 33413385 PMC7788718

[69] Suiyanka L Alegana VA Snow RW. Insecticide-treated net distribution in western kenya: impacts related to COVID-19 and health worker strikes. Int Health. (2021) 2021:ihab051. 10.1093/inthealth/ihab051 34401909 PMC8385957

[70] Wambua S Malla L Mbevi G Kandiah J Nwosu A-P Tuti T. Quantifying the indirect impact of COVID-19 pandemic on utilisation of outpatient and immunisation services in kenya: a longitudinal study using interrupted time series analysis. BMJ Open. (2022) 12:e055815. 10.1136/bmjopen-2021-055815 35273053 PMC8914407

[71] Noor AM Amin AA Gething PW Atkinson PM Hay SI Snow RW. Modelling distances travelled to government health services in Kenya. Trop Med Intern Health. (2006) 11:188–96. 10.1111/j.1365-3156.2005.01555.x 16451343 PMC2912494

[72] Bangha MW Simelane S. Spatial differentials in childhood mortality in south Africa: evidence from the 2001 census. Afri Popul Studies. (2007) 22:3–21. 10.11564/22-2-327

[73] Macharia PM Joseph NK Sartorius B Snow RW Okiro EA. Subnational estimates of factors associated with under-five mortality in kenya: a spatio-temporal analysis, 1993–2014. BMJ global health. (2021) 6:e004544. 10.1136/bmjgh-2020-004544 33858833 PMC8054106

[74] Noor A Zurovac D Hay S Ochola S Snow R. Defining equity in physical access to clinical services using geographical information systems as part of malaria planning and monitoring in kenya. Trop Med Int Health. (2003) 8:917–26. 10.1046/j.1365-3156.2003.01112.x 14516303 PMC2912492

[75] Chuma J Maina T Ataguba J. Does the distribution of health care benefits in kenya meet the principles of universal coverage? BMC Public Health. (2012) 12:20. 10.1186/1471-2458-12-20 22233470 PMC3280172

[76] Marek T O'Farrell C Yamamoto C Zable I. Trends and Opportunities in Public-Private Partnerships to Improve Health Service Delivery in Africa: Human Development Sector, Africa Region, The World Bank. Washington, DC: World Bank Group (2005).

[77] Organization(WHO) WH. The Private Sector, Universal Health Coverage and Primary Health Care. Geneva: World Health Organization (2018).

[78] Prata N Montagu D Jefferys E. Private sector, human resources and health franchising in Africa. Bull World Health Organ. (2005) 83:274–9. 15868018 PMC2626208

[79] Gatome-Munyua A Koseki S Chuma B Musau S Johns B. An Assessment of the Cost and Quality of Private Health Services in Kenya. Bethesda, MD: Strengthening Health Outcomes through the Private Sector Project, Abt Associates Inc (2015).

[80] Bazant ES Koenig MA Fotso JC Mills S. Women's use of private and government health facilities for childbirth in nairobi's. Inform Settlem Studies Family Plan. (2009) 40:39–50. 10.1111/j.1728-4465.2009.00185.x 19397184

[81] Banke-Thomas A Wong KL Collins L Olaniran A Balogun M Wright O. An assessment of geographical access and factors influencing travel time to emergency obstetric care in the urban state of lagos, nigeria. Health Policy Plan. (2021) 36:1384–96. 10.1093/heapol/czab099 34424314 PMC8505861

