# Supplementary material for: Geographic accessibility to public and private health facilities in Kenya in 2021: An updated geocoded inventory and spatial analysis
Source: Front Public Health. 2022 Nov 3;10:1002975. doi: 10.3389/fpubh.2022.1002975 (PMC9670107; doi:10.3389/fpubh.2022.1002975)
Supplement: Supplementary file 1 [file Data_Sheet_1.PDF]

Supplementary File 1

Table 1: Facilities excluded from updated facility on basis of not offering routine diagnosis and curative services

**Facility Type**	**Number**
Non-medical sites (medical stores, maintenance sites, blood depots, KEMSA, regulatory board offices, mortuaries, funeral homes, hospices etc)	25
Dental clinics	146
Ophthalmologists, opticians and eye Clinics	123
Rehab centres: orthopaedic, trauma, other	41
HIV/VCT and DICE	447
Family Planning centres	53
Special Clinics inc. physiotherapy, psychiatric, mental health, epilepsy, diabetes, TB, gynae, liver, ENT, dialysis, oncology, fertility, heart, chest, inoculation centres and other surgical	169
Labs & diagnostic centres (histology, X-ray, imagining, radiology urology, endoscopy, dermatology, infectious disease clinics)	209
Pharmacies	92
Non-Gen Pop (schools, farms, military, company clinics, hotels etc)	367

Figure 1: Counties and Projected Population in Kenya 2021

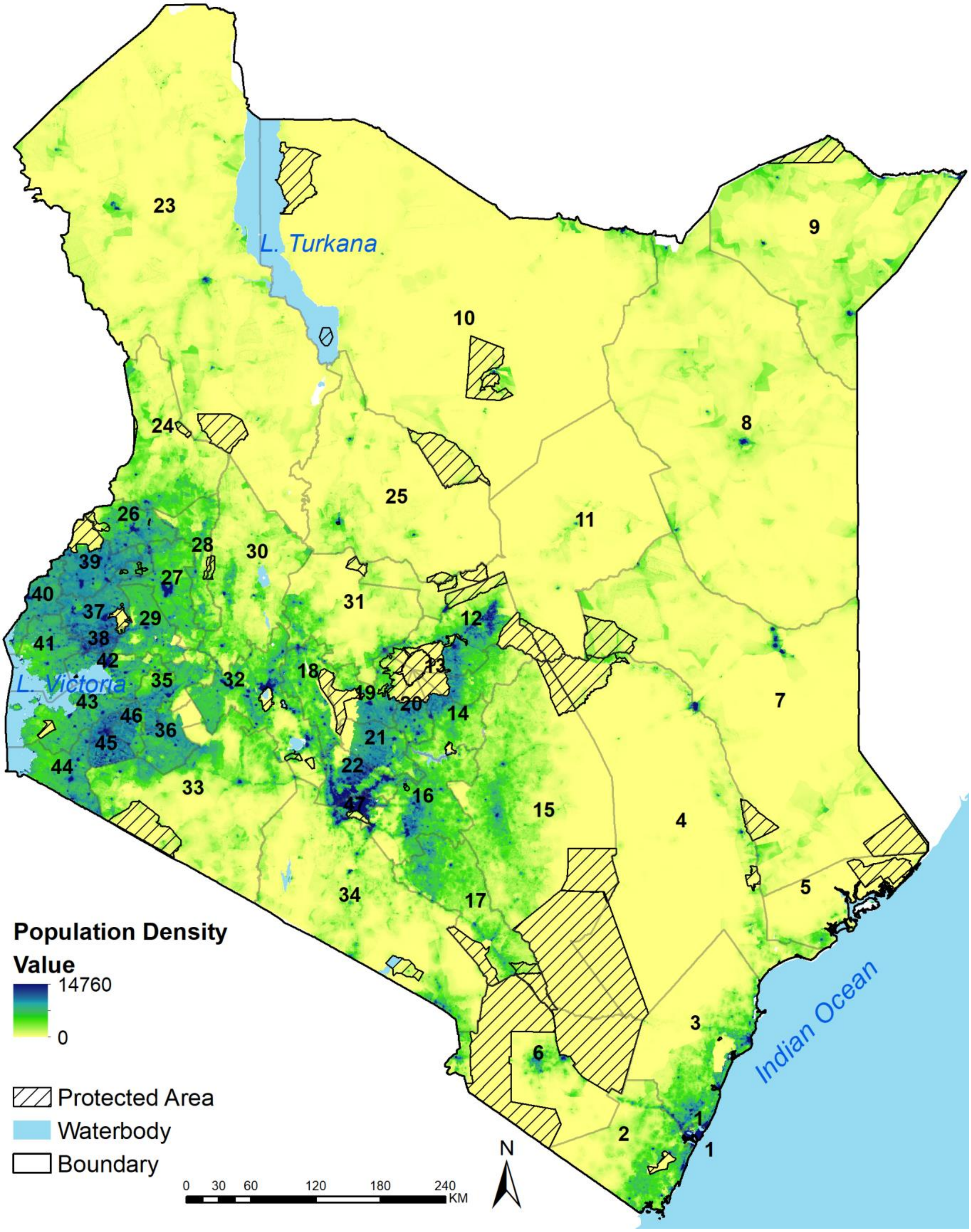

Mombasa [1], Kwale [2], Kilifi [3], Tana River [4], Lamu [5], Taita Taveta [6], Garissa[7], Wajir [8], Mandera [9], Marsabit [10], Isiolo [11], Meru [12], Tharaka-Nithi [13], Embu [14], Kitui [15], Machakos [16], Makueni [17], Nyandarua [18], Nyeri [19], Kirinyaga [20], Murang'a [21], Kiambu [22], Turkana [23], WestPokot [24], Samburu [25], Trans Nzoia [26], Uasin Gishu [27], Elgeyo-Marakwet [28], Nandi [29], Baringo[30],Laikipia [31], Nakuru [32], Narok [33], Kajiado [34],Kericho[35], Bomet [36], Kakamega [37], Vihiga [38], Bungoma[39], Busia [40], Siaya [41], Kisumu [42], Homa Bay [43], Migori [44], Kisii [45], Nyamira[46],Nairobi[47]

Figure 2: Road Network in Kenya

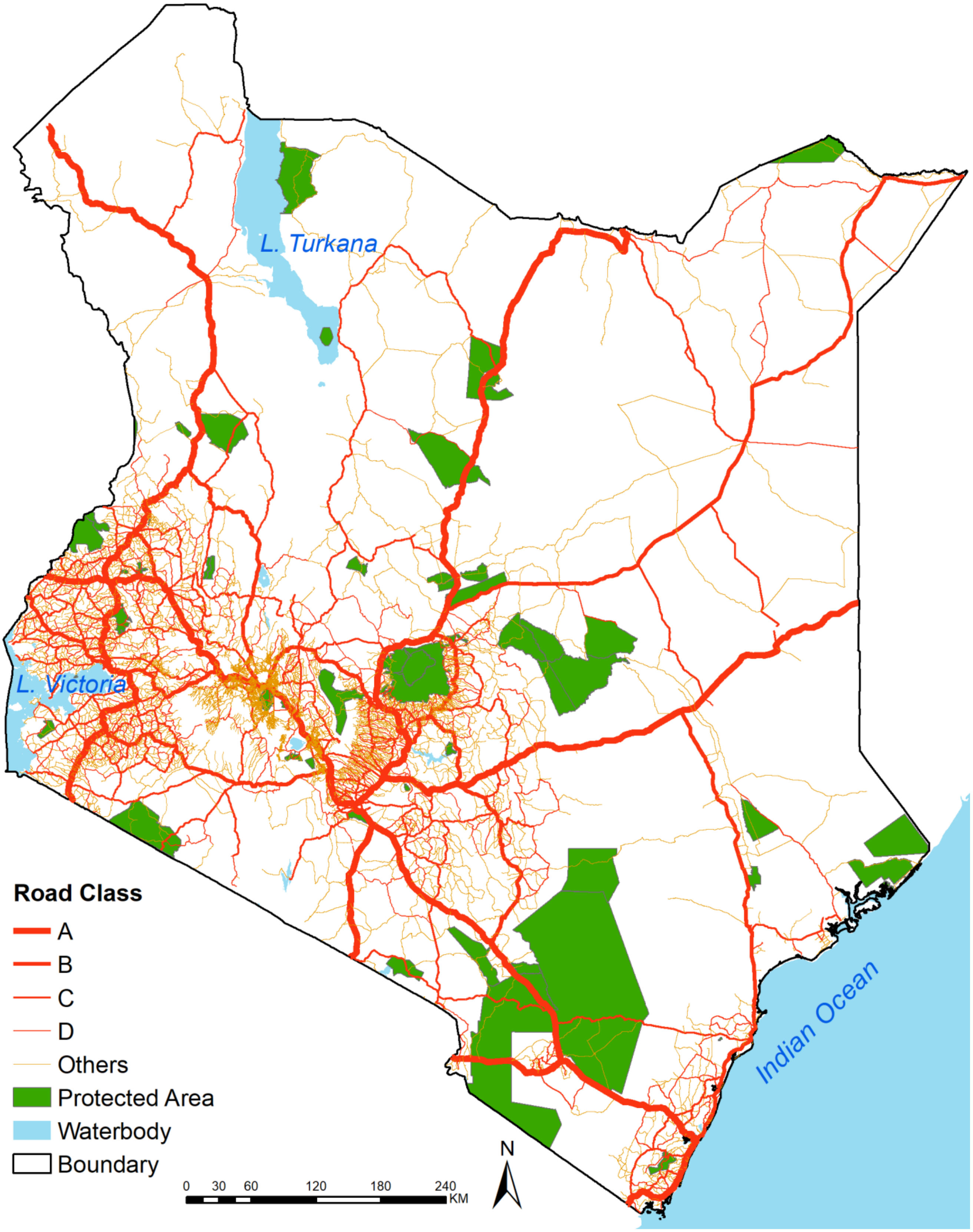

Figure 3:Land Use and Landcover Kenya 2021

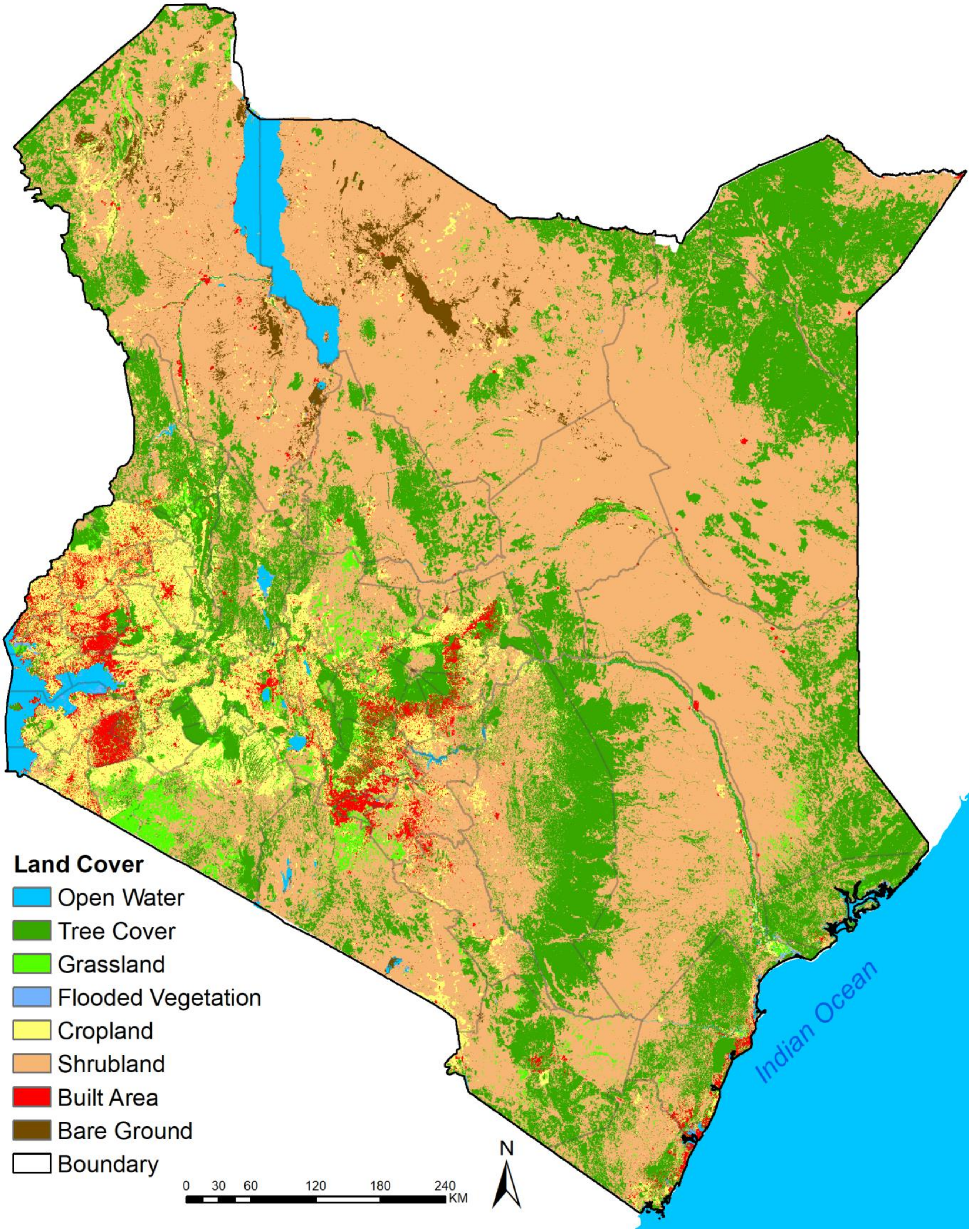

Figure 4: Digital Elevation Model Kenya

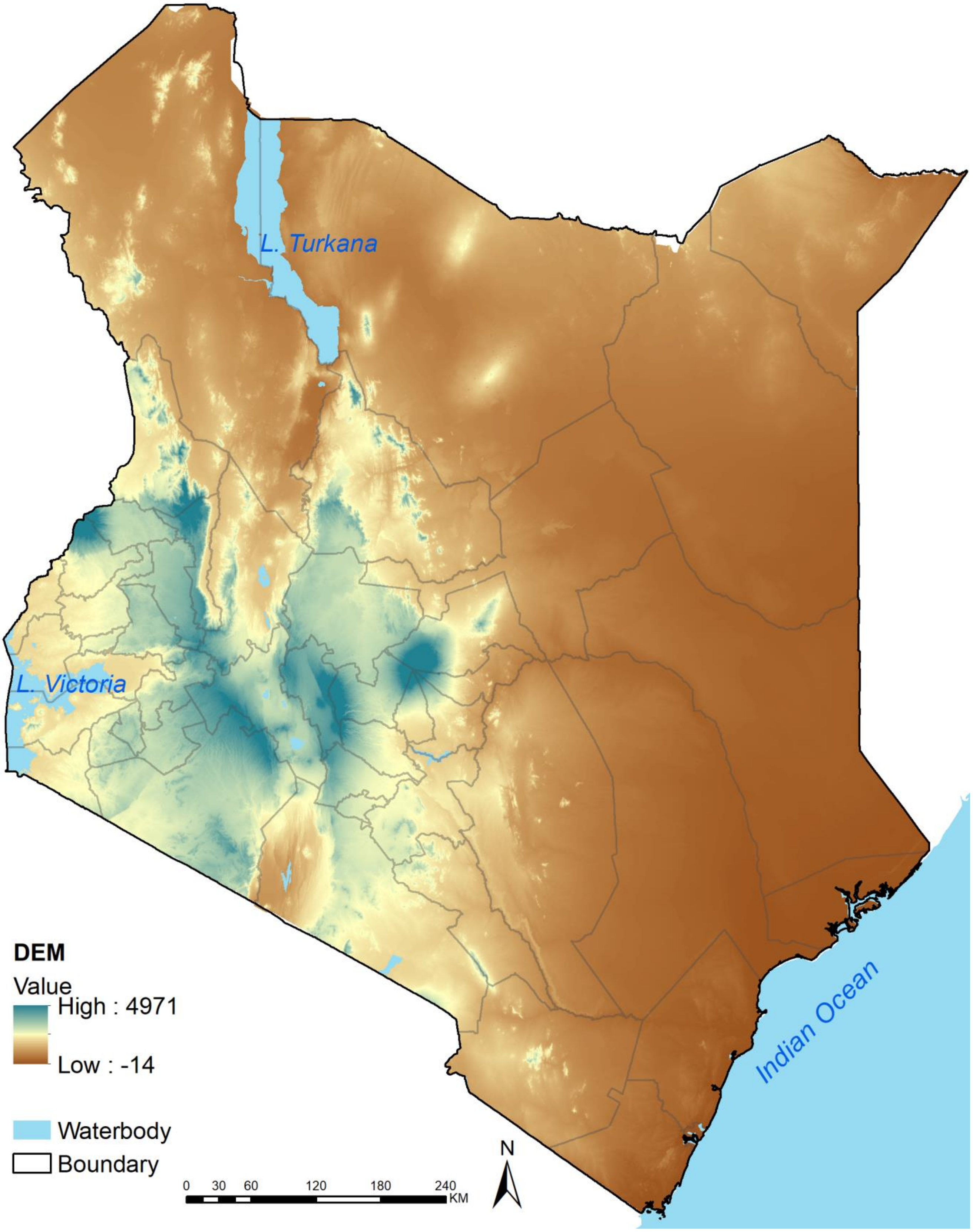

Figure 5: Transport Barriers

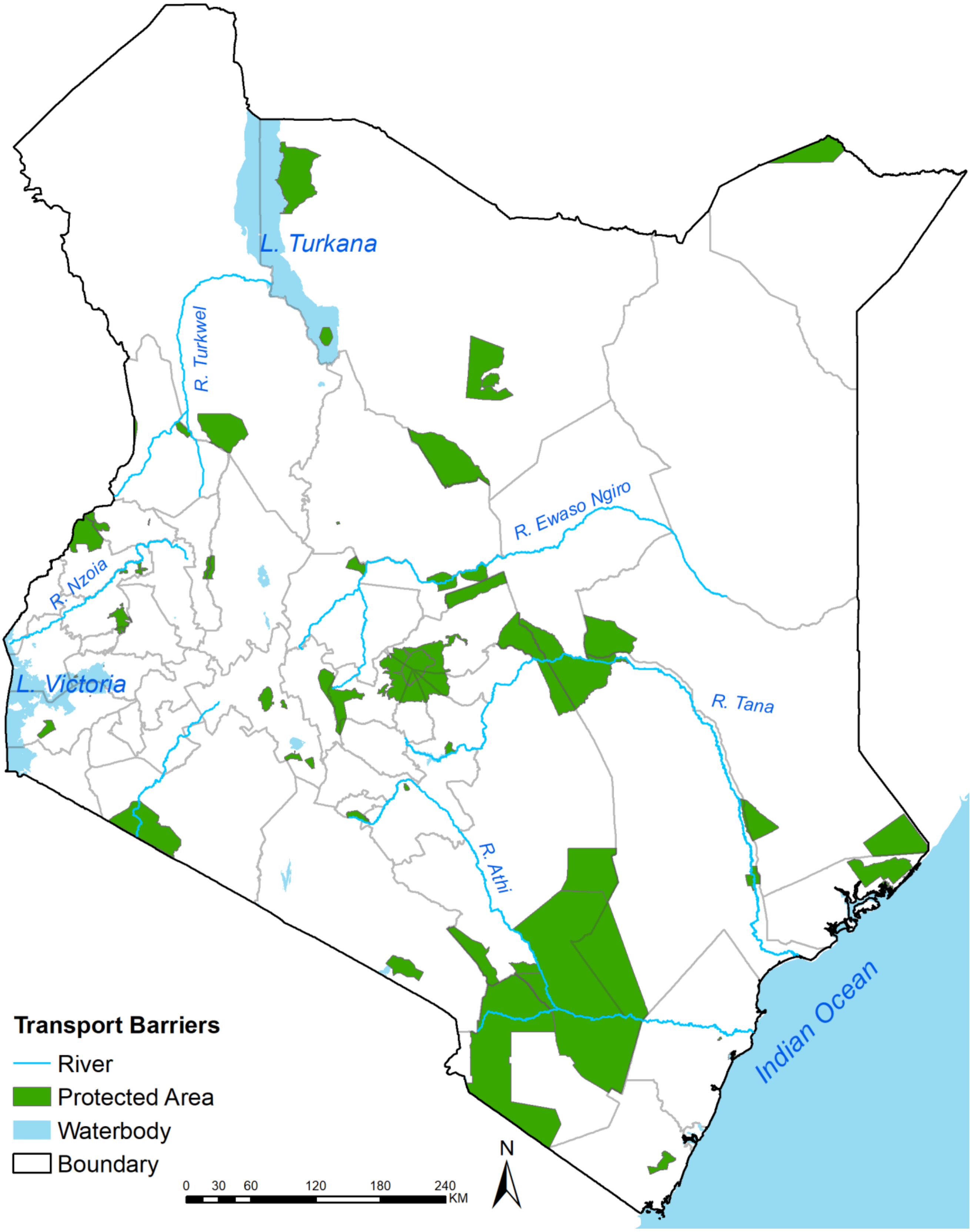

Figure 6: Flowchart summarising data assembly and analysis framework

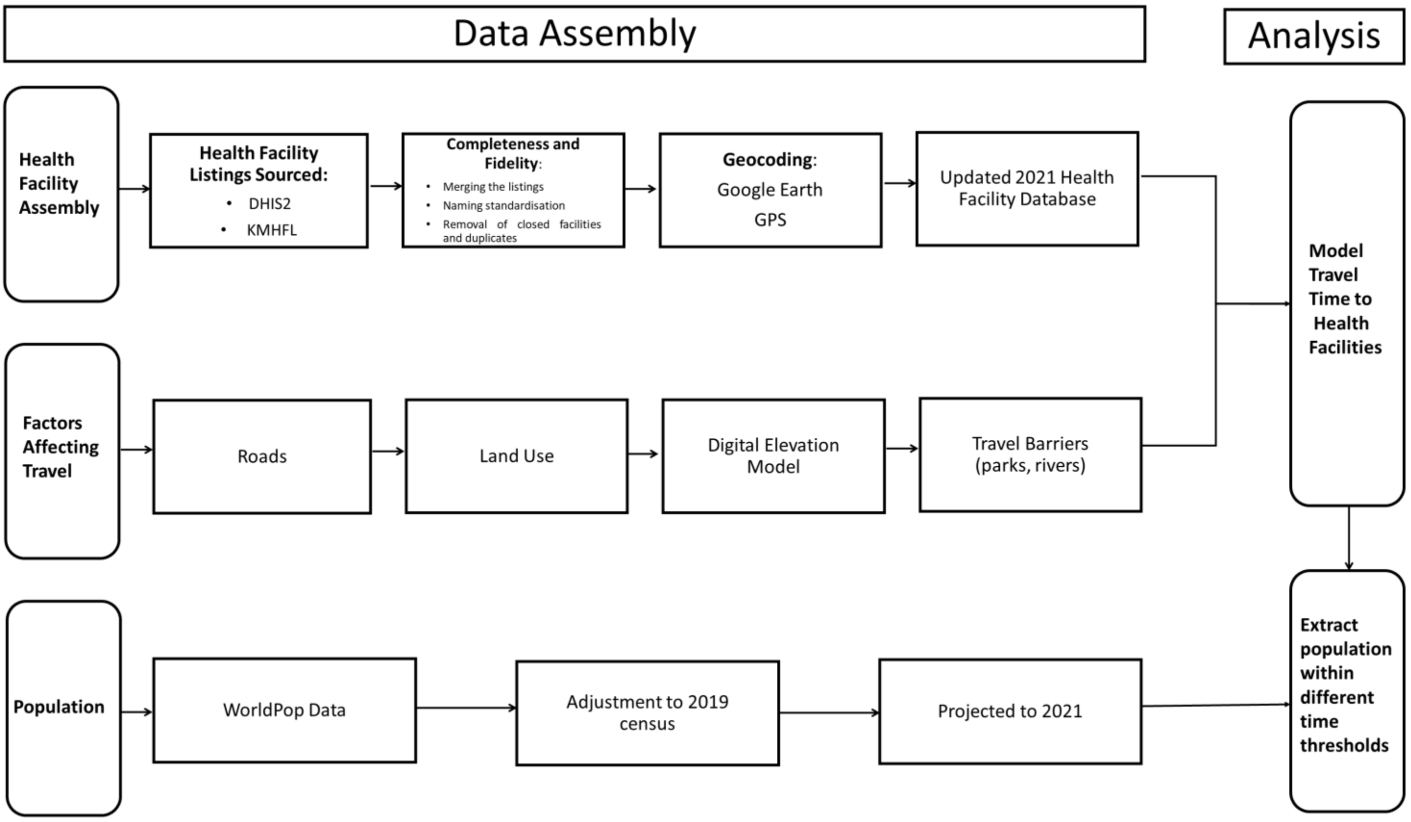

Figure 7: Proportion of population that is marginalized (beyond 2 hours) of a public, private, and combined (public and private) in 2021

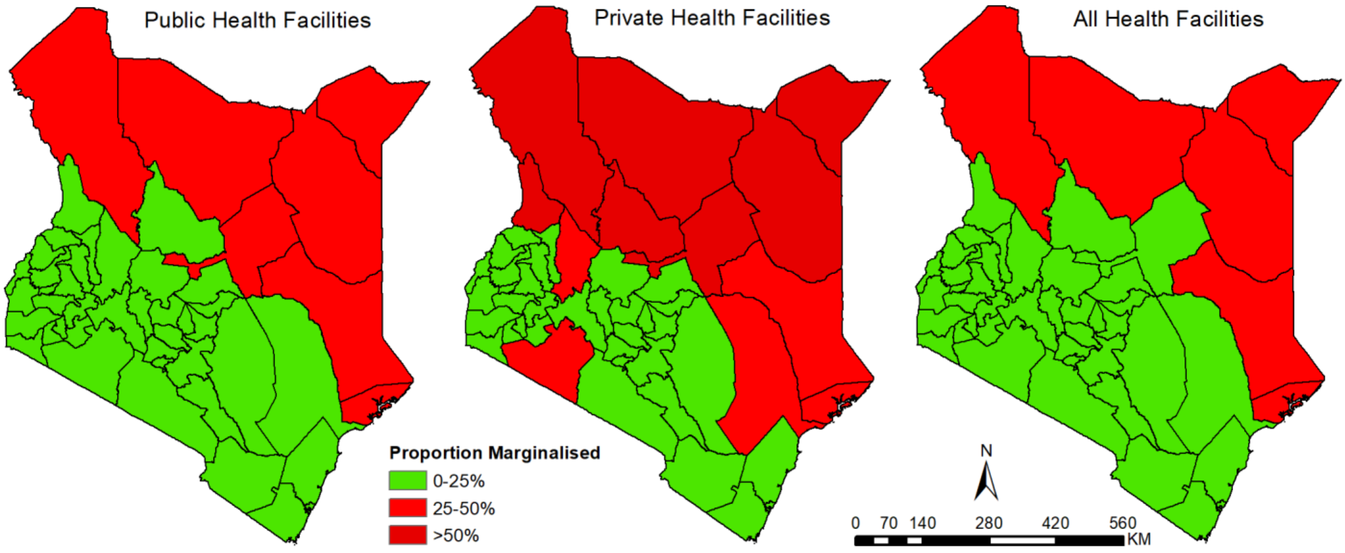

Table 2: Proportion of population within 1 hour of health facilities at the County level

	All Heath Facilities			Public (MoH, NGO, FBO)		Private	
**County**	**Population 2021**	**Population within 1 hour**	**(%)**	**Population within 1 hour**	**(%)**	**Population within 1 hour**	**(%)**
**Baringo**	682,009	521,766	**76.5**	520,624	**76.34**	277,996	**40.76**
**Bomet**	894,039	891,778	**99.75**	890,193	**99.57**	796,357	**89.07**
**Bungoma**	1,708,053	1,695,636	**99.27**	1,695,636	**99.27**	1,662,193	**97.32**
**Busia**	908,216	879,638	**96.85**	879,638	**96.85**	849,533	**93.54**
**Elgeyo-Marakwet**	465,775	414,464	**88.98**	414,351	**88.96**	261,038	**56.04**
**Embu**	621,610	592,033	**95.24**	591,337	**95.13**	551,918	**88.79**
**Garissa**	889,776	525,144	**59.02**	519,389	**58.37**	363,935	**40.9**
**Homa Bay**	1,149,042	1,079,682	**93.96**	1,073,114	**93.39**	976,848	**85.01**
**Isiolo**	299,426	167,615	**55.98**	166,943	**55.75**	117,371	**39.2**
**Kajiado**	1,212,592	965,531	**79.63**	955,161	**78.77**	823,068	**67.88**
**Kakamega**	1,880,578	1,855,132	**98.65**	1,854,654	**98.62**	1,816,703	**96.6**
**Kericho**	920,168	910,499	**98.95**	907,339	**98.61**	837,922	**91.06**
**Kiambu**	2,568,922	2,558,261	**99.59**	2,556,657	**99.52**	2,548,881	**99.22**
**Kilifi**	1,519,785	1,354,559	**89.13**	1,348,678	**88.74**	1,213,765	**79.86**
**Kirinyaga**	621,590	612,776	**98.58**	612,735	**98.58**	610,078	**98.15**
**Kisii**	1,270,101	1,270,005	**99.99**	1,270,005	**99.99**	1,261,101	**99.29**
**Kisumu**	1,180,552	1,162,412	**98.46**	1,162,307	**98.45**	1,133,068	**95.98**
**Kitui**	1,151,180	1,014,328	**88.11**	1,013,832	**88.07**	641,561	**55.73**
**Kwale**	909,741	785,893	**86.39**	780,985	**85.85**	636,918	**70.01**
**Laikipia**	539,700	459,457	**85.13**	453,144	**83.96**	371,203	**68.78**
**Lamu**	125,538	69,203	**55.13**	68,379	**54.47**	49,882	**39.73**
**Machakos**	1,480,086	1,448,273	**97.85**	1,443,306	**97.52**	1,279,562	**86.45**
**Makueni**	999,313	943,479	**94.41**	942,143	**94.28**	640,674	**64.11**
**Mandera**	840,519	343,136	**40.82**	338,983	**40.33**	252,513	**30.04**
**Marsabit**	496,888	204,769	**41.21**	204,745	**41.21**	128,013	**25.76**
**Meru**	1,573,642	1,494,520	**94.97**	1,490,149	**94.69**	1,407,200	**89.42**
**Migori**	1,139,735	1,117,669	**98.06**	1,117,669	**98.06**	1,037,299	**91.01**
**Mombasa**	1,227,439	1,061,460	**86.48**	1,060,872	**86.43**	1,055,543	**86**
**Murang a**	1,066,146	1,061,831	**99.6**	1,061,765	**99.59**	1,052,849	**98.75**
**Nairobi**	4,646,550	4,635,148	**99.75**	4,634,590	**99.74**	4,633,483	**99.72**
**Nakuru**	2,265,377	2,206,950	**97.42**	2,204,592	**97.32**	2,090,548	**92.28**
**Nandi**	901,786	881,755	**97.78**	881,406	**97.74**	695,304	**77.1**
**Narok**	1,210,330	865,522	**71.51**	856,794	**70.79**	520,334	**42.99**
**Nyamira**	599,514	599,458	**99.99**	599,458	**99.99**	598,946	**99.91**
**Nyandarua**	638,831	631,026	**98.78**	630,131	**98.64**	618,034	**96.74**
**Nyeri**	762,947	733,223	**96.1**	731,876	**95.93**	729,281	**95.59**
**Samburu**	327,010	175,575	**53.69**	173,051	**52.92**	83,198	**25.44**
**Siaya**	1,008,728	977,683	**96.92**	977,524	**96.91**	956,034	**94.78**
**Taita Taveta**	348,313	300,566	**86.29**	298,599	**85.73**	266,663	**76.56**
**Tana River**	329,248	176,915	**53.73**	173,419	**52.67**	93,954	**28.54**
**Tharaka-Nithi**	394,711	378,544	**95.9**	378,075	**95.79**	331,208	**83.91**
**Trans Nzoia**	1,012,641	982,151	**96.99**	981,626	**96.94**	907,366	**89.6**
**Turkana**	926,193	352,087	**38.01**	350,237	**37.81**	179,283	**19.36**
**Uasin Gishu**	1,204,894	1,195,754	**99.24**	1,195,398	**99.21**	1,098,512	**91.17**
**Vihiga**	588,514	582,818	**99.03**	582,818	**99.03**	582,802	**99.03**
**Wajir**	805,273	313,806	**38.97**	307,412	**38.17**	191,374	**23.77**
**West Pokot**	635,337	383,320	**60.33**	383,226	**60.32**	171,309	**26.96**
**National**	**48,948,358**	**43,833,250**	**89.55**	**43,734,965**	**89.35**	**39,402,625**	**80.5**

Table 3: Proportion of population within 2 hours of health facilities at the County Level

County	All Heath Facilities			Public (MoH, NGO, FBO)		Private	
	Population 2021	Population within 2 hours	(%)	Population within 2 hours	(%)	Population within 2 hours	(%)
**Baringo**	682,009	639,332	**93.74**	638,853	**93.67**	469,674	**68.87**
**Bomet**	894,039	893,460	**99.94**	893,460	**99.94**	890,894	**99.65**
**Bungoma**	1,708,053	1,699,718	**99.51**	1,699,718	**99.51**	1,698,952	**99.47**
**Busia**	908,216	880,879	**96.99**	880,879	**96.99**	879,795	**96.87**
**Elgeyo-Marakwet**	465,775	454,809	**97.65**	454,809	**97.65**	410,737	**88.18**
**Embu**	621,610	610,411	**98.2**	610,411	**98.2**	600,235	**96.56**
**Garissa**	889,776	658,846	**74.05**	650,466	**73.1**	491,193	**55.2**
**Homa Bay**	1,149,042	1,094,647	**95.27**	1,093,734	**95.19**	1,087,013	**94.6**
**Isiolo**	299,426	224,667	**75.03**	224,407	**74.95**	140,571	**46.95**
**Kajiado**	1,212,592	1,136,345	**93.71**	1,132,683	**93.41**	1,012,892	**83.53**
**Kakamega**	1,880,578	1,856,178	**98.7**	1,856,178	**98.7**	1,856,178	**98.7**
**Kericho**	920,168	919,083	**99.88**	918,857	**99.86**	914,503	**99.38**
**Kiambu**	2,568,922	2,562,133	**99.74**	2,562,126	**99.74**	2,562,012	**99.73**
**Kilifi**	1,519,785	1,425,690	**93.81**	1,424,749	**93.75**	1,373,094	**90.35**
**Kirinyaga**	621,590	614,627	**98.88**	614,627	**98.88**	614,627	**98.88**
**Kisii**	1,270,101	1,270,005	**99.99**	1,270,005	**99.99**	1,270,005	**99.99**
**Kisumu**	1,180,552	1,165,044	**98.69**	1,165,044	**98.69**	1,165,044	**98.69**
**Kitui**	1,151,180	1,118,320	**97.15**	1,118,284	**97.14**	947,026	**82.27**
**Kwale**	909,741	855,542	**94.04**	851,439	**93.59**	822,363	**90.4**
**Laikipia**	539,700	519,444	**96.25**	517,245	**95.84**	473,745	**87.78**
**Lamu**	125,538	86,033	**68.53**	85,171	**67.84**	77,728	**61.92**
**Machakos**	1,480,086	1,462,632	**98.82**	1,462,432	**98.81**	1,452,785	**98.16**
**Makueni**	999,313	980,410	**98.11**	980,337	**98.1**	881,993	**88.26**
**Mandera**	840,519	525,689	**62.54**	520,654	**61.94**	392,667	**46.72**
**Marsabit**	496,888	292,070	**58.78**	292,053	**58.78**	195,784	**39.4**
**Meru**	1,573,642	1,537,905	**97.73**	1,537,905	**97.73**	1,525,891	**96.97**
**Migori**	1,139,735	1,117,905	**98.08**	1,117,905	**98.08**	1,117,019	**98.01**
**Mombasa**	1,227,439	1,062,107	**86.53**	1,062,107	**86.53**	1,062,107	**86.53**
**Murang a**	1,066,146	1,062,872	**99.69**	1,062,872	**99.69**	1,062,872	**99.69**
**Nairobi**	4,646,550	4,635,296	**99.76**	4,635,296	**99.76**	4,635,296	**99.76**
**Nakuru**	2,265,377	2,238,432	**98.81**	2,238,302	**98.8**	2,219,872	**97.99**
**Nandi**	901,786	896,845	**99.45**	896,820	**99.45**	891,522	**98.86**
**Narok**	1,210,330	1,120,281	**92.56**	1,119,178	**92.47**	841,307	**69.51**
**Nyamira**	599,514	599,458	**99.99**	599,458	**99.99**	599,458	**99.99**
**Nyandarua**	638,831	635,463	**99.47**	635,463	**99.47**	634,811	**99.37**
**Nyeri**	762,947	735,643	**96.42**	735,643	**96.42**	734,836	**96.32**
**Samburu**	327,010	251,940	**77.04**	251,483	**76.9**	142,254	**43.5**
**Siaya**	1,008,728	980,625	**97.21**	980,625	**97.21**	976,765	**96.83**
**Taita Taveta**	348,313	326,693	**93.79**	325,669	**93.5**	314,676	**90.34**
**Tana River**	329,248	250,265	**76.01**	248,008	**75.33**	169,229	**51.4**
**Tharaka-Nithi**	394,711	392,053	**99.33**	392,053	**99.33**	379,335	**96.1**
**Trans Nzoia**	1,012,641	1,001,382	**98.89**	1,001,309	**98.88**	997,490	**98.5**
**Turkana**	926,193	575,935	**62.18**	573,401	**61.91**	295,463	**31.9**
**Uasin Gishu**	1,204,894	1,200,774	**99.66**	1,200,774	**99.66**	1,199,049	**99.51**
**Vihiga**	588,514	582,818	**99.03**	582,818	**99.03**	582,818	**99.03**
**Wajir**	805,273	519,955	**64.57**	512,305	**63.62**	309,501	**38.43**
**West Pokot**	635,337	545,815	**85.91**	545,802	**85.91**	294,438	**46.34**
**National**	**48,948,358**	**46,216,476**	**94.42**	**46,173,817**	**94.33**	**43,667,519**	**89.21**

Table 4: Proportion of population within 1 hour of health facilities at the Sub County Level

County	Sub County	All Heath Facilities			Public (MoH, NGO, FBO)		Private	
		Population 2021	Population within 2 hours	(%)	Population within 2 hours	(%)	Population within 2 hours	(%)
**Baringo**	**Baringo Central**	104,937	96,965	92	96,965	92	72,602	69
**Baringo**	**Baringo North**	113,540	87,904	77	87,167	77	21,140	19
**Baringo**	**Baringo South**	97,258	78,989	81	78,964	81	52,631	54
**Baringo**	**Eldama Ravine**	148,018	140,996	95	140,915	95	110,455	75
**Baringo**	**Mogotio**	53,573	42,436	79	42,136	79	16,812	31
**Baringo**	**Tiaty**	164,682	74,476	45	74,476	45	4,357	3
**Bomet**	**Bomet Central**	141,188	141,188	100	141,188	100	130,525	92
**Bomet**	**Bomet East**	158,710	158,710	100	158,710	100	122,858	77
**Bomet**	**Chepalungu**	205,115	205,084	100	205,084	100	168,565	82
**Bomet**	**Konoin**	176,294	174,151	99	172,566	98	163,617	93
**Bomet**	**Sotik**	212,733	212,644	100	212,644	100	210,791	99
**Bungoma**	**Bumula**	223,924	223,924	100	223,924	100	222,959	100
**Bungoma**	**Kabuchai**	178,707	178,707	100	178,707	100	178,707	100
**Bungoma**	**Kanduyi**	294,716	294,304	100	294,304	100	293,918	100
**Bungoma**	**Kimilili**	162,781	162,781	100	162,781	100	162,781	100
**Bungoma**	**Mt. Elgon**	193,616	183,670	95	183,670	95	158,657	82
**Bungoma**	**Sirisia**	129,389	127,515	99	127,515	99	126,848	98
**Bungoma**	**Tongaren**	233,612	233,486	100	233,486	100	228,087	98
**Bungoma**	**Webuye East**	146,674	146,674	100	146,674	100	146,608	100
**Bungoma**	**Webuye West**	144,634	144,575	100	144,575	100	143,628	99
**Busia**	**Nambale**	117,750	117,750	100	117,750	100	117,186	100
**Busia**	**Teso North**	138,266	132,095	96	132,095	96	124,426	90
**Busia**	**Bunyala**	83,560	76,423	91	76,423	91	68,459	82
**Busia**	**Butula**	151,259	151,259	100	151,259	100	146,431	97
**Busia**	**Samia**	111,839	107,740	96	107,740	96	106,618	95
**Busia**	**Matayos**	150,029	142,062	95	142,062	95	141,699	94
**Busia**	**Teso South**	155,512	152,310	98	152,310	98	144,714	93
**Elgeyo-Marakwet**	**Keiyo North**	95,031	88,891	94	88,891	94	65,403	69
**Elgeyo-Marakwet**	**Keiyo South**	138,120	130,967	95	130,889	95	93,038	67
**Elgeyo-Marakwet**	**Marakwet East**	106,838	76,071	71	76,036	71	43,107	40
**Elgeyo-Marakwet**	**Marakwet West**	125,786	118,535	94	118,535	94	59,491	47
**Embu**	**Manyatta**	215,385	213,310	99	213,310	99	213,310	99
**Embu**	**Mbeere North**	106,479	99,042	93	98,703	93	85,451	80
**Embu**	**Mbeere South**	158,528	141,283	89	140,925	89	114,758	72
**Embu**	**Runyenjes**	141,218	138,398	98	138,398	98	138,398	98
**Garissa**	**Balambala**	80,272	11,102	14	11,023	14	838	1
**Garissa**	**Dadaab**	227,956	174,106	76	174,017	76	152,130	67
**Garissa**	**Fafi**	179,877	108,532	60	107,592	60	25,268	14
**Garissa**	**Garissa**	163,293	156,878	96	156,813	96	156,711	96
**Garissa**	**Ijara**	103,634	30,115	29	25,563	25	8,387	8
**Garissa**	**Lagdera**	134,743	44,411	33	44,381	33	20,600	15
**Homa Bay**	**Homa Bay**	111,466	105,713	95	105,713	95	105,566	95
**Homa Bay**	**Rachuonyo East**	130,021	129,032	99	129,032	99	128,739	99
**Homa Bay**	**Rachuonyo North**	190,387	179,898	94	179,898	94	174,077	91
**Homa Bay**	**Rachuonyo South**	133,936	133,936	100	133,936	100	131,138	98
**Homa Bay**	**Suba North**	136,560	106,673	78	100,672	74	94,367	69
**Homa Bay**	**Ndhiwa**	203,363	199,335	98	199,303	98	158,849	78
**Homa Bay**	**Rangwe**	121,253	120,480	99	120,480	99	116,700	96
**Homa Bay**	**Suba South**	122,056	104,615	86	104,079	85	67,413	55
**Isiolo**	**Garbatulla**	97,191	39,162	40	39,106	40	9,274	10
**Isiolo**	**Merti**	44,994	15,032	33	14,954	33	7,967	18
**Kajiado**	**Kajiado Central**	185,861	123,621	67	122,428	66	89,530	48
**Kajiado**	**Kajiado East**	223,843	192,940	86	189,377	85	172,880	77
**Kajiado**	**Kajiado North**	346,973	345,878	100	345,878	100	345,878	100
**Kajiado**	**Kajiado South**	242,242	174,001	72	171,567	71	112,470	46
**Kajiado**	**Kajiado West**	213,673	129,091	60	125,910	59	102,311	48
**Kakamega**	**Likuyani**	145,539	141,329	97	141,308	97	141,173	97

**Kakamega**	**Lugari**	189,057	178,030	94	178,030	94	176,360	93
**Kakamega**	**Matungu**	166,783	166,744	100	166,744	100	163,469	98
**Kakamega**	**Navakholo**	156,754	155,404	99	155,404	99	145,549	93
**Kakamega**	**Butere**	158,207	158,207	100	158,207	100	158,207	100
**Kakamega**	**Ikolomani**	117,742	117,200	100	117,200	100	117,200	100
**Kakamega**	**Khwisero**	112,394	111,745	99	111,745	99	111,745	99
**Kakamega**	**Lurambi**	178,632	178,632	100	178,632	100	178,632	100
**Kakamega**	**Malava**	230,124	229,694	100	229,694	100	219,562	95
**Kakamega**	**Mumias East**	125,386	124,933	100	124,933	100	114,604	91
**Kakamega**	**Mumias West**	112,673	111,006	99	110,549	98	110,281	98
**Kakamega**	**Shinyalu**	187,288	182,208	97	182,208	97	179,919	96
**Kericho**	**Kipkelion East**	146,475	141,984	97	140,815	96	128,747	88
**Kericho**	**Belgut**	169,680	168,493	99	167,770	99	167,013	98
**Kericho**	**Bureti**	198,963	198,393	100	198,393	100	198,393	100
**Kericho**	**Soin-Sigowet**	124,347	123,711	99	123,711	99	105,127	85
**Kericho**	**Ainamoi**	167,659	167,439	100	167,439	100	161,503	96
**Kericho**	**Kipkelion West**	113,044	110,478	98	109,210	97	77,137	68
**Kiambu**	**Kikuyu**	194,118	193,528	100	193,378	100	193,442	100
**Kiambu**	**Limuru**	209,293	208,222	99	208,030	99	207,195	99
**Kiambu**	**Gatundu North**	170,645	170,397	100	170,397	100	170,076	100
**Kiambu**	**Gatundu South**	168,944	168,798	100	168,794	100	168,713	100
**Kiambu**	**Githunguri**	243,649	243,486	100	243,486	100	243,486	100
**Kiambu**	**Juja**	202,501	199,721	99	199,447	98	197,150	97
**Kiambu**	**Kabete**	191,157	191,157	100	191,157	100	191,157	100
**Kiambu**	**Kiambaa**	198,943	198,890	100	198,890	100	198,890	100
**Kiambu**	**Kiambu**	175,457	175,082	100	175,082	100	175,082	100
**Kiambu**	**Lari**	203,992	202,019	99	201,881	99	198,146	97
**Kiambu**	**Ruiru**	340,912	339,336	100	338,489	99	338,997	99
**Kiambu**	**Thika Town**	269,312	267,627	99	267,627	99	266,548	99
**Kilifi**	**Ganze**	171,883	135,600	79	132,627	77	74,025	43
**Kilifi**	**Kaloleni**	218,593	212,572	97	212,378	97	183,875	84
**Kilifi**	**Kilifi North**	294,108	259,527	88	259,271	88	253,014	86
**Kilifi**	**Kilifi South**	248,686	239,382	96	239,159	96	233,103	94
**Kilifi**	**Magarini**	217,613	161,196	74	158,986	73	139,952	64
**Kilifi**	**Malindi**	238,252	216,290	91	216,264	91	200,970	84
**Kilifi**	**Rabai**	130,649	129,993	99	129,993	99	128,827	99
**Kirinyaga**	**Kirinyaga East**	140,637	136,428	97	136,428	97	136,428	97
**Kirinyaga**	**Mwea East**	129,788	127,606	98	127,565	98	124,908	96
**Kirinyaga**	**Mwea West**	110,490	110,304	100	110,304	100	110,304	100
**Kirinyaga**	**Kirinyaga Central**	127,611	125,483	98	125,483	98	125,483	98
**Kirinyaga**	**Kirinyaga West**	113,064	112,954	100	112,954	100	112,954	100
**Kisii**	**Nyaribari Masaba**	135,664	135,664	100	135,664	100	135,364	100
**Kisii**	**Bonchari**	124,254	124,254	100	124,254	100	124,254	100
**Kisii**	**Kitutu Chache North**	119,152	119,152	100	119,152	100	119,152	100
**Kisii**	**Kitutu Chache South**	114,309	114,309	100	114,309	100	114,309	100
**Kisii**	**Bobasi**	213,319	213,319	100	213,319	100	213,319	100
**Kisii**	**Bomachoge Borabu**	119,232	119,232	100	119,232	100	119,137	100
**Kisii**	**Bomachoge Chache**	99,425	99,425	100	99,425	100	99,425	100
**Kisii**	**Nyaribari Chache**	169,569	169,569	100	169,569	100	169,569	100
**Kisii**	**South Mugirango**	175,177	175,081	100	175,081	100	166,572	95
**Kisumu**	**Nyakach**	152,105	145,690	96	145,690	96	142,807	94
**Kisumu**	**Nyando**	173,092	170,159	98	170,159	98	164,718	95
**Kisumu**	**Seme**	123,259	122,656	100	122,656	100	113,832	92
**Kisumu**	**Muhoroni**	170,702	170,300	100	170,195	100	158,760	93
**Kisumu**	**Kisumu Central**	216,443	211,176	98	211,176	98	211,176	98
**Kisumu**	**Kisumu East**	178,841	177,373	99	177,373	99	176,717	99
**Kisumu**	**Kisumu West**	166,110	165,058	99	165,058	99	165,058	99
**Kitui**	**Mwingi Central**	187,495	167,716	89	167,690	89	85,673	46
**Kitui**	**Mwingi North**	159,546	119,232	75	118,939	75	34,213	21
**Kitui**	**Mwingi West**	93,150	91,908	99	91,908	99	71,861	77
**Kitui**	**Kitui Central**	115,556	112,593	97	112,593	97	84,803	73
**Kitui**	**Kitui East**	136,669	110,220	81	110,117	81	71,981	53
**Kitui**	**Kitui Rural**	152,276	145,961	96	145,918	96	110,571	73
**Kitui**	**Kitui South**	193,905	154,289	80	154,258	80	91,086	47
**Kitui**	**Kitui West**	112,583	112,410	100	112,410	100	91,373	81
**Kwale**	**Kinango**	304,630	241,460	79	238,725	78	190,337	62
**Kwale**	**Matuga**	201,834	185,835	92	185,745	92	131,800	65

**Kwale**	**Msambweni**	193,617	188,089	97	187,520	97	187,515	97
**Kwale**	**Lunga Lunga**	209,660	170,508	81	168,996	81	127,266	61
**Laikipia**	**Laikipia North**	119,245	74,544	63	72,931	61	36,915	31
**Laikipia**	**Laikipia West**	288,771	260,644	90	259,538	90	225,332	78
**Laikipia**	**Laikipia East**	131,684	124,269	94	120,674	92	108,955	83
**Lamu**	**Lamu East**	11,264	1,827	16	1,827	16	-	-
**Lamu**	**Lamu West**	114,274	67,376	59	66,552	58	49,882	44
**Machakos**	**Masinga**	165,494	154,162	93	153,915	93	93,720	57
**Machakos**	**Machakos**	268,799	267,694	100	267,694	100	250,106	93
**Machakos**	**Mavoko**	198,751	194,985	98	192,740	97	193,254	97
**Machakos**	**Matungulu**	167,839	159,719	95	159,199	95	152,195	91
**Machakos**	**Yatta**	199,711	195,162	98	193,268	97	160,360	80
**Machakos**	**Mwala**	221,891	219,116	99	219,054	99	173,113	78
**Machakos**	**Kangundo**	121,779	121,779	100	121,779	100	121,157	99
**Machakos**	**Kathiani**	135,822	135,657	100	135,657	100	135,657	100
**Makueni**	**Kibwezi East**	147,764	113,679	77	113,630	77	75,677	51
**Makueni**	**Kibwezi West**	186,788	176,275	94	175,409	94	122,664	66
**Makueni**	**Kilome**	104,889	102,956	98	102,945	98	93,256	89
**Makueni**	**Makueni**	214,423	209,531	98	209,216	98	105,670	49
**Makueni**	**Mbooni**	213,313	209,258	98	209,237	98	123,525	58
**Makueni**	**Kaiti**	132,137	131,780	100	131,706	100	119,881	91
**Mandera**	**Banissa**	112,300	30,180	27	30,156	27	14,557	13
**Mandera**	**Lafey**	104,744	27,162	26	27,069	26	9,537	9
**Mandera**	**Mandera East**	132,149	82,599	63	82,174	62	72,237	55
**Mandera**	**Mandera North**	135,745	57,041	42	56,525	42	37,288	27
**Mandera**	**Mandera West**	143,306	49,852	35	47,525	33	35,336	25
**Mandera**	**Kutullo**	65,846	14,005	21	14,005	21	6,718	10
**Mandera**	**Mandera South**	146,429	82,297	56	81,529	56	76,840	52
**Marsabit**	**Laisamis**	111,942	26,717	24	26,717	24	5,569	5
**Marsabit**	**North Horr**	127,235	19,957	16	19,957	16	5,021	4
**Marsabit**	**Moyale**	178,656	111,522	62	111,497	62	78,191	44
**Marsabit**	**Saku**	79,055	46,574	59	46,574	59	39,233	50
**Meru**	**Buuri**	161,087	150,427	93	149,385	93	146,248	91
**Meru**	**Central Imenti**	157,578	153,353	97	152,550	97	134,185	85
**Meru**	**Igembe Central**	226,510	213,382	94	213,231	94	187,335	83
**Meru**	**Igembe North**	169,064	143,421	85	142,207	84	134,149	79
**Meru**	**Igembe South**	149,394	148,052	99	147,750	99	145,124	97
**Meru**	**Tigania East**	195,476	179,538	92	178,699	91	164,775	84
**Meru**	**Tigania West**	143,174	142,990	100	142,983	100	134,221	94
**Meru**	**North Imenti**	160,414	154,002	96	154,002	96	153,974	96
**Meru**	**South Imenti**	210,945	209,355	99	209,343	99	207,191	98
**Migori**	**Awendo**	134,702	134,702	100	134,702	100	131,477	98
**Migori**	**Nyatike**	177,114	165,141	93	165,141	93	136,034	77
**Migori**	**Rongo**	128,639	128,639	100	128,639	100	128,061	100
**Migori**	**Uriri**	144,001	143,857	100	143,857	100	122,049	85
**Migori**	**Kuria East**	130,955	128,241	98	128,241	98	117,304	90
**Migori**	**Kuria West**	185,680	180,257	97	180,257	97	178,896	96
**Migori**	**Suna East**	123,939	123,741	100	123,741	100	120,873	98
**Migori**	**Suna West**	114,705	113,091	99	113,091	99	102,605	89
**Mombasa**	**Jomvu**	158,518	138,801	88	138,527	87	138,801	88
**Mombasa**	**Kisauni**	300,224	272,124	91	271,809	91	266,614	89
**Mombasa**	**Changamwe**	162,363	142,878	88	142,878	88	142,878	88
**Mombasa**	**Likoni**	191,955	149,155	78	149,155	78	148,748	77
**Mombasa**	**Mvita**	164,027	121,064	74	121,064	74	121,064	74
**Mombasa**	**Nyali**	250,352	237,439	95	237,439	95	237,439	95
**Murang'a**	**Maragwa**	173,209	171,431	99	171,431	99	169,656	98
**Murang'a**	**Gatanga**	190,811	189,090	99	189,090	99	182,585	96
**Murang'a**	**Kiharu**	105,448	105,327	100	105,327	100	105,327	100
**Murang'a**	**Kahuro**	68,418	68,418	100	68,418	100	68,418	100
**Murang'a**	**Kandara**	174,566	174,566	100	174,566	100	174,566	100
**Murang'a**	**Kangema**	117,100	116,918	100	116,918	100	116,492	99
**Murang'a**	**Kigumo**	136,752	136,699	100	136,699	100	136,423	100
**Murang'a**	**Mathioya**	99,842	99,381	100	99,315	99	99,381	100
**Nairobi**	**Dagoretti South**	260,022	259,502	100	259,502	100	259,502	100
**Nairobi**	**Langata**	271,314	264,130	97	264,130	97	264,130	97
**Nairobi**	**Kasarani**	304,905	302,439	99	301,881	99	300,775	99
**Nairobi**	**Roysambu**	258,067	257,741	100	257,741	100	257,741	100
**Nairobi**	**Westlands**	281,853	281,416	100	281,416	100	281,416	100
**Nairobi**	**Embakasi East**	370,535	370,301	100	370,301	100	370,301	100
**Nairobi**	**Embakasi South**	245,691	245,455	100	245,455	100	245,455	100

**Nairobi**	**Dagoretti North**	286,175	286,175	100	286,175	100	286,175	100
**Nairobi**	**Embakasi Central**	285,513	285,513	100	285,513	100	285,513	100
**Nairobi**	**Embakasi North**	286,819	286,819	100	286,819	100	286,819	100
**Nairobi**	**Embakasi West**	300,481	300,481	100	300,481	100	300,481	100
**Nairobi**	**Kamukunji**	267,712	267,712	100	267,712	100	267,712	100
**Nairobi**	**Kibra**	265,066	265,066	100	265,066	100	265,066	100
**Nairobi**	**Makadara**	235,028	235,028	100	235,028	100	235,028	100
**Nairobi**	**Mathare**	316,597	316,597	100	316,597	100	316,597	100
**Nairobi**	**Ruaraka**	202,127	202,127	100	202,127	100	202,127	100
**Nairobi**	**Starehe**	208,646	208,646	100	208,646	100	208,646	100
**Nakuru**	**Kuresoi North**	183,283	182,009	99	181,980	99	159,088	87
**Nakuru**	**Molo**	172,830	169,871	98	169,712	98	164,957	95
**Nakuru**	**Rongai**	186,998	181,825	97	181,818	97	166,646	89
**Nakuru**	**Subukia**	121,821	120,320	99	120,312	99	114,066	94
**Nakuru**	**Kuresoi South**	153,420	148,524	97	148,432	97	101,796	66
**Nakuru**	**Naivasha**	325,193	300,044	92	299,520	92	294,657	91
**Nakuru**	**Bahati**	203,943	202,543	99	202,223	99	199,935	98
**Nakuru**	**Gilgil**	204,621	199,032	97	198,098	97	195,428	96
**Nakuru**	**Nakuru Town East**	257,471	257,191	100	257,191	100	257,190	100
**Nakuru**	**Nakuru Town West**	200,009	199,573	100	199,573	100	199,558	100
**Nakuru**	**Njoro**	255,789	246,017	96	245,733	96	237,228	93
**Nandi**	**Chesumei**	144,537	142,681	99	142,681	99	124,680	86
**Nandi**	**Emgwen**	162,934	160,225	98	160,225	98	142,201	87
**Nandi**	**Mosop**	156,416	156,331	100	156,331	100	134,372	86
**Nandi**	**Tinderet**	123,779	116,408	94	116,207	94	47,940	39
**Nandi**	**Aldai**	185,334	182,366	98	182,218	98	144,653	78
**Nandi**	**Nandi Hills**	128,785	123,744	96	123,744	96	101,458	79
**Narok**	**Transmara East**	127,874	127,836	100	127,836	100	86,063	67
**Narok**	**Transmara West**	271,926	228,697	84	225,255	83	158,763	58
**Narok**	**Narok North**	265,190	191,151	72	187,576	71	140,985	53
**Narok**	**Narok South**	260,647	127,828	49	126,815	49	43,380	17
**Narok**	**Narok West**	190,116	113,187	60	112,903	59	41,623	22
**Narok**	**Narok East**	94,576	76,824	81	76,410	81	49,519	52
**Nyamira**	**Borabu**	80,635	80,601	100	80,601	100	80,089	99
**Nyamira**	**Nyamira North**	160,956	160,935	100	160,935	100	160,935	100
**Nyamira**	**Nyamira South**	157,408	157,408	100	157,408	100	157,408	100
**Nyamira**	**Masaba North**	108,975	108,975	100	108,975	100	108,975	100
**Nyamira**	**Manga**	91,539	91,539	100	91,539	100	91,539	100
**Nyandarua**	**Kinangop**	202,334	201,034	99	200,892	99	200,302	99
**Nyandarua**	**Ndaragwa**	93,319	90,597	97	90,485	97	83,104	89
**Nyandarua**	**Ol Joro Orok**	114,645	113,828	99	113,415	99	113,796	99
**Nyandarua**	**Kipipiri**	102,512	99,926	97	99,698	97	98,490	96
**Nyandarua**	**Ol Kalou**	126,022	125,642	100	125,642	100	122,342	97
**Nyeri**	**Mathira East**	105,873	102,430	97	102,430	97	102,430	97
**Nyeri**	**Mukurweini**	93,491	93,491	100	93,491	100	93,491	100
**Nyeri**	**Kieni East**	115,568	96,517	84	95,243	82	96,487	83
**Nyeri**	**Kieni West**	86,369	82,929	96	82,856	96	79,154	92
**Nyeri**	**Mathira West**	54,991	52,372	95	52,372	95	52,372	95
**Nyeri**	**Othaya**	96,324	95,815	99	95,815	99	95,815	99
**Nyeri**	**Tetu**	82,570	82,072	99	82,072	99	81,935	99
**Nyeri**	**Nyeri Central**	127,760	127,597	100	127,597	100	127,597	100
**Samburu**	**Samburu West**	121,401	91,852	76	89,611	74	59,447	49
**Samburu**	**Isiolo**	157,241	113,421	72	112,883	72	100,131	64
**Samburu**	**Samburu East**	84,687	36,322	43	36,260	43	20,839	25
**Samburu**	**Samburu North**	120,922	47,402	39	47,180	39	2,913	2
**Siaya**	**Alego-usonga**	225,869	220,627	98	220,627	98	216,728	96
**Siaya**	**Bondo**	184,590	166,131	90	165,973	90	153,878	83
**Siaya**	**Ugenya**	130,261	130,135	100	130,135	100	129,902	100
**Siaya**	**Rarieda**	164,552	159,024	97	159,024	97	154,963	94
**Siaya**	**Gem**	193,523	193,197	100	193,197	100	191,995	99
**Siaya**	**Ugunja**	109,933	108,568	99	108,568	99	108,568	99
**Taita Taveta**	**Taveta**	82,540	75,734	92	74,206	90	67,271	82
**Taita Taveta**	**Voi**	113,863	92,531	81	92,359	81	82,735	73
**Taita Taveta**	**Mwatate**	79,597	70,231	88	70,146	88	63,332	80
**Taita Taveta**	**Wundanyi**	72,313	62,069	86	61,888	86	53,325	74
**Tana River**	**Bura**	139,795	67,547	48	67,451	48	30,469	22
**Tana River**	**Galole**	77,977	47,928	61	46,100	59	26,566	34
**Tana River**	**Garsen**	111,477	61,440	55	59,868	54	36,919	33
**Tharaka-Nithi**	**Tharaka South**	77,251	69,799	90	69,513	90	44,197	57

**Tharaka-Nithi**	**Maara**	125,583	125,018	100	124,932	99	123,649	98
**Tharaka-Nithi**	**Tharaka North**	54,636	47,457	87	47,457	87	27,572	50
**Tharaka-Nithi**	**Chuka/Igambang ombe**	137,241	136,271	99	136,173	99	135,791	99
**Trans Nzoia**	**Endebess**	111,597	96,085	86	95,828	86	73,165	66
**Trans Nzoia**	**Kiminini**	242,545	242,513	100	242,513	100	238,640	98
**Trans Nzoia**	**Saboti**	229,837	220,855	96	220,740	96	215,752	94
**Trans Nzoia**	**Cherangany**	248,557	246,126	99	246,022	99	236,187	95
**Trans Nzoia**	**Kwanza**	180,105	176,571	98	176,522	98	143,622	80
**Turkana**	**Turkana East**	100,571	18,890	19	18,890	19	9,876	10
**Turkana**	**Turkana Central**	138,828	79,821	57	79,784	57	58,710	42
**Turkana**	**Turkana North**	148,143	42,984	29	42,984	29	1,737	1
**Turkana**	**Loima**	145,039	49,719	34	49,707	34	12,570	9
**Turkana**	**Turkana South**	145,304	64,375	44	64,375	44	23,941	16
**Turkana**	**Turkana West**	248,307	96,298	39	94,498	38	72,449	29
**Uasin Gishu**	**Ainabkoi**	139,436	139,018	100	138,991	100	122,894	88
**Uasin Gishu**	**Kesses**	199,289	193,490	97	193,430	97	190,280	95
**Uasin Gishu**	**Moiben**	179,055	178,744	100	178,531	100	148,565	83
**Uasin Gishu**	**Soy**	225,989	225,378	100	225,363	100	192,404	85
**Uasin Gishu**	**Turbo**	259,057	257,495	99	257,495	99	255,764	99
**Uasin Gishu**	**Kapseret**	202,069	201,629	100	201,588	100	188,605	93
**Vihiga**	**Emuhaya**	90,952	90,952	100	90,952	100	90,952	100
**Vihiga**	**Hamisi**	153,113	147,416	96	147,416	96	147,400	96
**Vihiga**	**Sabatia**	135,032	135,032	100	135,032	100	135,032	100
**Vihiga**	**Luanda**	104,456	104,456	100	104,456	100	104,456	100
**Vihiga**	**Vihiga**	104,962	104,962	100	104,962	100	104,962	100
**Wajir**	**Wajir South**	165,023	47,637	29	47,506	29	23,976	15
**Wajir**	**Eldas**	96,374	30,212	31	29,600	31	14,698	15
**Wajir**	**Wajir West**	102,673	36,398	35	36,354	35	17,822	17
**Wajir**	**Tarbaj**	116,715	36,506	31	33,512	29	7,332	6
**Wajir**	**Wajir East**	141,202	105,173	74	105,035	74	101,208	72
**Wajir**	**Wajir North**	183,286	57,881	32	55,404	30	26,339	14
**West Pokot**	**Central Pokot**	108,470	48,464	45	48,464	45	11,234	10
**West Pokot**	**Pokot South**	162,327	123,335	76	123,267	76	68,956	42
**West Pokot**	**West Pokot**	169,622	130,484	77	130,459	77	91,119	54
**West Pokot**	**North Pokot**	194,918	81,037	42	81,037	42	-	-

Table 5: Proportion of population within 2 hours of health facilities at the Sub County Level

County	Sub County	All Heath Facilities			Public (MoH, NGO,FBO)		Private	
		Population 2021	Population within 2 hours	(%)	Population within 2 hours	(%)	Population within 2 hours	(%)
**Baringo**	**Baringo Central**	104,937	104,795	100	104,795	100	99,724	95
**Baringo**	**Baringo North**	113,540	106,221	94	105,984	93	65,109	57
**Baringo**	**Baringo South**	97,258	92,166	95	92,166	95	84,314	87
**Baringo**	**Eldama Ravine**	148,018	147,177	99	147,165	99	144,052	97
**Baringo**	**Mogotio**	53,573	52,813	99	52,584	98	43,995	82
**Baringo**	**Tiaty**	164,682	136,160	83	136,160	83	32,481	20
**Bomet**	**Bomet Central**	141,188	141,188	100	141,188	100	141,188	100
**Bomet**	**Bomet East**	158,710	158,710	100	158,710	100	156,389	99
**Bomet**	**Chepalungu**	205,115	205,115	100	205,115	100	205,115	100
**Bomet**	**Konoin**	176,294	175,715	100	175,715	100	175,469	100
**Bomet**	**Sotik**	212,733	212,733	100	212,733	100	212,733	100
**Bungoma**	**Bumula**	223,924	223,924	100	223,924	100	223,924	100
**Bungoma**	**Kabuchai**	178,707	178,707	100	178,707	100	178,707	100
**Bungoma**	**Kanduyi**	294,716	294,304	100	294,304	100	294,304	100
**Bungoma**	**Kimilili**	162,781	162,781	100	162,781	100	162,781	100
**Bungoma**	**Mt. Elgon**	193,616	187,753	97	187,753	97	186,987	97
**Bungoma**	**Sirisia**	129,389	127,515	99	127,515	99	127,515	99
**Bungoma**	**Tongaren**	233,612	233,486	100	233,486	100	233,486	100
**Bungoma**	**Webuye East**	146,674	146,674	100	146,674	100	146,674	100
**Bungoma**	**Webuye West**	144,634	144,575	100	144,575	100	144,575	100
**Busia**	**Nambale**	117,750	117,750	100	117,750	100	117,750	100
**Busia**	**Teso North**	138,266	132,246	96	132,246	96	132,246	96
**Busia**	**Bunyala**	83,560	77,432	93	77,432	93	76,348	91
**Busia**	**Butula**	151,259	151,259	100	151,259	100	151,259	100
**Busia**	**Samia**	111,839	107,770	96	107,770	96	107,770	96
**Busia**	**Matayos**	150,029	142,062	95	142,062	95	142,062	95
**Busia**	**Teso South**	155,512	152,360	98	152,360	98	152,360	98
**Elgeyo-Marakwet**	**Keiyo North**	95,031	92,268	97	92,268	97	85,354	90
**Elgeyo-Marakwet**	**Keiyo South**	138,120	137,963	100	137,963	100	130,255	94
**Elgeyo-Marakwet**	**Marakwet East**	106,838	99,318	93	99,318	93	77,118	72
**Elgeyo-Marakwet**	**Marakwet West**	125,786	125,260	100	125,260	100	118,010	94
**Embu**	**Manyatta**	215,385	213,310	99	213,310	99	213,310	99
**Embu**	**Mbeere North**	106,479	106,389	100	106,389	100	100,404	94
**Embu**	**Mbeere South**	158,528	152,314	96	152,314	96	148,123	93
**Embu**	**Runyenjes**	141,218	138,398	98	138,398	98	138,398	98
**Garissa**	**Balambala**	80,272	29,785	37	29,676	37	5,490	7
**Garissa**	**Dadaab**	227,956	196,479	86	196,450	86	170,568	75
**Garissa**	**Fafi**	179,877	130,925	73	127,732	71	95,764	53
**Garissa**	**Garissa**	163,293	160,695	98	160,654	98	159,443	98
**Garissa**	**Ijara**	103,634	61,123	59	56,127	54	18,164	18
**Garissa**	**Lagdera**	134,743	79,838	59	79,826	59	41,765	31
**Homa Bay**	**Homa Bay**	111,466	105,713	95	105,713	95	105,713	95
**Homa Bay**	**Rachuonyo East**	130,021	129,032	99	129,032	99	129,032	99
**Homa Bay**	**Rachuonyo North**	190,387	180,194	95	180,194	95	180,194	95
**Homa Bay**	**Rachuonyo South**	133,936	133,936	100	133,936	100	133,936	100
**Homa Bay**	**Suba North**	136,560	110,598	81	109,705	80	110,134	81
**Homa Bay**	**Ndhiwa**	203,363	202,765	100	202,765	100	202,765	100
**Homa Bay**	**Rangwe**	121,253	120,480	99	120,480	99	120,480	99
**Homa Bay**	**Suba South**	122,056	111,928	92	111,908	92	104,758	86
**Isiolo**	**Garbatulla**	97,191	65,357	67	65,356	67	19,693	20
**Isiolo**	**Merti**	44,994	25,907	58	25,897	58	13,472	30
**Kajiado**	**Kajiado Central**	185,861	169,636	91	169,212	91	126,685	68
**Kajiado**	**Kajiado East**	223,843	222,489	99	222,149	99	212,230	95
**Kajiado**	**Kajiado North**	346,973	345,878	100	345,878	100	345,878	100
**Kajiado**	**Kajiado South**	242,242	220,896	91	219,232	91	189,822	78
**Kajiado**	**Kajiado West**	213,673	177,446	83	176,213	82	138,277	65

**Kakamega**	**Likuyani**	145,539	141,329	97	141,329	97	141,329	97
**Kakamega**	**Lugari**	189,057	178,030	94	178,030	94	178,030	94
**Kakamega**	**Matungu**	166,783	166,744	100	166,744	100	166,744	100
**Kakamega**	**Navakholo**	156,754	155,404	99	155,404	99	155,404	99
**Kakamega**	**Butere**	158,207	158,207	100	158,207	100	158,207	100
**Kakamega**	**Ikolomani**	117,742	117,200	100	117,200	100	117,200	100
**Kakamega**	**Khwisero**	112,394	111,745	99	111,745	99	111,745	99
**Kakamega**	**Lurambi**	178,632	178,632	100	178,632	100	178,632	100
**Kakamega**	**Malava**	230,124	229,694	100	229,694	100	229,694	100
**Kakamega**	**Mumias East**	125,386	125,128	100	125,128	100	125,128	100
**Kakamega**	**Mumias West**	112,673	111,856	99	111,856	99	111,856	99
**Kakamega**	**Shinyalu**	187,288	182,208	97	182,208	97	182,208	97
**Kericho**	**Kipkelion East**	146,475	146,335	100	146,288	100	145,182	99
**Kericho**	**Belgut**	169,680	169,390	100	169,335	100	169,327	100
**Kericho**	**Bureti**	198,963	198,393	100	198,393	100	198,393	100
**Kericho**	**Soin-Sigowet**	124,347	124,289	100	124,289	100	123,861	100
**Kericho**	**Ainamoi**	167,659	167,659	100	167,659	100	167,659	100
**Kericho**	**Kipkelion West**	113,044	113,016	100	112,892	100	110,081	97
**Kiambu**	**Kikuyu**	194,118	193,819	100	193,819	100	193,819	100
**Kiambu**	**Limuru**	209,293	209,095	100	209,095	100	209,095	100
**Kiambu**	**Gatundu North**	170,645	170,530	100	170,525	100	170,530	100
**Kiambu**	**Gatundu South**	168,944	168,944	100	168,944	100	168,944	100
**Kiambu**	**Githunguri**	243,649	243,486	100	243,486	100	243,486	100
**Kiambu**	**Juja**	202,501	199,721	99	199,721	99	199,721	99
**Kiambu**	**Kabete**	191,157	191,157	100	191,157	100	191,157	100
**Kiambu**	**Kiambaa**	198,943	198,890	100	198,890	100	198,890	100
**Kiambu**	**Kiambu**	175,457	175,082	100	175,082	100	175,082	100
**Kiambu**	**Lari**	203,992	203,686	100	203,684	100	203,566	100
**Kiambu**	**Ruiru**	340,912	340,097	100	340,097	100	340,097	100
**Kiambu**	**Thika Town**	269,312	267,627	99	267,627	99	267,627	99
**Kilifi**	**Ganze**	171,883	167,933	98	167,687	98	143,751	84
**Kilifi**	**Kaloleni**	218,593	218,167	100	218,167	100	215,712	99
**Kilifi**	**Kilifi North**	294,108	262,987	89	262,984	89	262,881	89
**Kilifi**	**Kilifi South**	248,686	241,722	97	241,713	97	241,722	97
**Kilifi**	**Magarini**	217,613	185,485	85	184,801	85	162,696	75
**Kilifi**	**Malindi**	238,252	218,925	92	218,925	92	215,860	91
**Kilifi**	**Rabai**	130,649	130,472	100	130,472	100	130,472	100
**Kirinyaga**	**Kirinyaga East**	140,637	136,428	97	136,428	97	136,428	97
**Kirinyaga**	**Mwea East**	129,788	129,457	100	129,457	100	129,457	100
**Kirinyaga**	**Mwea West**	110,490	110,304	100	110,304	100	110,304	100
**Kirinyaga**	**Kirinyaga Central**	127,611	125,483	98	125,483	98	125,483	98
**Kirinyaga**	**Kirinyaga West**	113,064	112,954	100	112,954	100	112,954	100
**Kisii**	**Nyaribari Masaba**	135,664	135,664	100	135,664	100	135,664	100
**Kisii**	**Bonchari**	124,254	124,254	100	124,254	100	124,254	100
**Kisii**	**Kitutu Chache North**	119,152	119,152	100	119,152	100	119,152	100
**Kisii**	**Kitutu Chache South**	114,309	114,309	100	114,309	100	114,309	100
**Kisii**	**Bobasi**	213,319	213,319	100	213,319	100	213,319	100
**Kisii**	**Bomachoge Borabu**	119,232	119,232	100	119,232	100	119,232	100
**Kisii**	**Bomachoge Chache**	99,425	99,425	100	99,425	100	99,425	100
**Kisii**	**Nyaribari Chache**	169,569	169,569	100	169,569	100	169,569	100
**Kisii**	**South Mugirango**	175,177	175,081	100	175,081	100	175,081	100
**Kisumu**	**Nyakach**	152,105	146,427	96	146,427	96	146,427	96
**Kisumu**	**Nyando**	173,092	171,662	99	171,662	99	171,662	99
**Kisumu**	**Seme**	123,259	122,656	100	122,656	100	122,656	100
**Kisumu**	**Muhoroni**	170,702	170,428	100	170,428	100	170,428	100
**Kisumu**	**Kisumu Central**	216,443	211,176	98	211,176	98	211,176	98
**Kisumu**	**Kisumu East**	178,841	177,559	99	177,559	99	177,559	99
**Kisumu**	**Kisumu West**	166,110	165,136	99	165,136	99	165,136	99
**Kitui**	**Mwingi Central**	187,495	183,026	98	183,026	98	134,129	72
**Kitui**	**Mwingi North**	159,546	149,092	93	149,092	93	96,128	60
**Kitui**	**Mwingi West**	93,150	93,044	100	93,044	100	88,408	95
**Kitui**	**Kitui Central**	115,556	115,329	100	115,329	100	114,091	99
**Kitui**	**Kitui East**	136,669	130,329	95	130,292	95	110,660	81
**Kitui**	**Kitui Rural**	152,276	151,977	100	151,977	100	141,627	93
**Kitui**	**Kitui South**	193,905	182,984	94	182,984	94	149,798	77
**Kitui**	**Kitui West**	112,583	112,538	100	112,538	100	112,185	100
**Kwale**	**Kinango**	304,630	277,588	91	276,467	91	262,005	86

**Kwale**	**Matuga**	201,834	197,032	98	197,032	98	191,251	95
**Kwale**	**Msambweni**	193,617	189,073	98	188,781	98	189,073	98
**Kwale**	**Lunga Lunga**	209,660	191,850	92	189,159	90	180,034	86
**Laikipia**	**Laikipia North**	119,245	104,741	88	103,857	87	68,964	58
**Laikipia**	**Laikipia West**	288,771	284,068	98	282,758	98	276,042	96
**Laikipia**	**Laikipia East**	131,684	130,635	99	130,631	99	128,739	98
**Lamu**	**Lamu East**	11,264	2,590	23	2,590	23	12	0
**Lamu**	**Lamu West**	114,274	83,443	73	82,581	72	77,716	68
**Machakos**	**Masinga**	165,494	157,457	95	157,457	95	153,996	93
**Machakos**	**Machakos**	268,799	268,433	100	268,432	100	268,290	100
**Machakos**	**Mavoko**	198,751	197,777	100	197,578	99	197,777	100
**Machakos**	**Matungulu**	167,839	161,608	96	161,608	96	161,604	96
**Machakos**	**Yatta**	199,711	198,895	100	198,895	100	198,522	99
**Machakos**	**Mwala**	221,891	221,026	100	221,026	100	215,160	97
**Machakos**	**Kangundo**	121,779	121,779	100	121,779	100	121,779	100
**Machakos**	**Kathiani**	135,822	135,657	100	135,657	100	135,657	100
**Makueni**	**Kibwezi East**	147,764	130,570	88	130,570	88	105,136	71
**Makueni**	**Kibwezi West**	186,788	186,117	100	186,044	100	166,022	89
**Makueni**	**Kilome**	104,889	104,789	100	104,789	100	104,789	100
**Makueni**	**Makueni**	214,423	214,220	100	214,220	100	168,718	79
**Makueni**	**Mbooni**	213,313	212,602	100	212,602	100	205,215	96
**Makueni**	**Kaiti**	132,137	132,113	100	132,113	100	132,113	100
**Mandera**	**Banissa**	112,300	54,889	49	54,869	49	33,716	30
**Mandera**	**Lafey**	104,744	59,265	57	59,075	56	23,462	22
**Mandera**	**Mandera East**	132,149	95,268	72	95,060	72	84,153	64
**Mandera**	**Mandera North**	135,745	87,669	65	87,541	64	63,151	47
**Mandera**	**Mandera West**	143,306	89,935	63	85,831	60	68,156	48
**Mandera**	**Kutullo**	65,846	30,040	46	30,040	46	20,752	32
**Mandera**	**Mandera South**	146,429	108,623	74	108,238	74	99,276	68
**Marsabit**	**Laisamis**	111,942	51,415	46	51,415	46	17,871	16
**Marsabit**	**North Horr**	127,235	44,277	35	44,274	35	13,884	11
**Marsabit**	**Moyale**	178,656	140,583	79	140,569	79	118,823	67
**Marsabit**	**Saku**	79,055	55,795	71	55,795	71	45,207	57
**Meru**	**Buuri**	161,087	153,688	95	153,688	95	152,897	95
**Meru**	**Central Imenti**	157,578	157,251	100	157,251	100	157,061	100
**Meru**	**Igembe Central**	226,510	223,166	99	223,166	99	218,403	96
**Meru**	**Igembe North**	169,064	160,842	95	160,842	95	156,059	92
**Meru**	**Igembe South**	149,394	149,041	100	149,041	100	149,041	100
**Meru**	**Tigania East**	195,476	187,571	96	187,571	96	186,083	95
**Meru**	**Tigania West**	143,174	142,990	100	142,990	100	142,990	100
**Meru**	**North Imenti**	160,414	154,002	96	154,002	96	154,002	96
**Meru**	**South Imenti**	210,945	209,355	99	209,355	99	209,355	99
**Migori**	**Awendo**	134,702	134,702	100	134,702	100	134,702	100
**Migori**	**Nyatike**	177,114	165,212	93	165,212	93	164,326	93
**Migori**	**Rongo**	128,639	128,639	100	128,639	100	128,639	100
**Migori**	**Uriri**	144,001	143,857	100	143,857	100	143,857	100
**Migori**	**Kuria East**	130,955	128,241	98	128,241	98	128,241	98
**Migori**	**Kuria West**	185,680	180,257	97	180,257	97	180,257	97
**Migori**	**Suna East**	123,939	123,741	100	123,741	100	123,741	100
**Migori**	**Suna West**	114,705	113,256	99	113,256	99	113,256	99
**Mombasa**	**Jomvu**	158,518	138,801	88	138,801	88	138,801	88
**Mombasa**	**Kisauni**	300,224	272,696	91	272,696	91	272,696	91
**Mombasa**	**Changamwe**	162,363	142,878	88	142,878	88	142,878	88
**Mombasa**	**Likoni**	191,955	149,230	78	149,230	78	149,230	78
**Mombasa**	**Mvita**	164,027	121,064	74	121,064	74	121,064	74
**Mombasa**	**Nyali**	250,352	237,439	95	237,439	95	237,439	95
**Murang'a**	**Maragwa**	173,209	172,413	100	172,413	100	172,413	100
**Murang'a**	**Gatanga**	190,811	189,124	99	189,124	99	189,124	99
**Murang'a**	**Kiharu**	105,448	105,327	100	105,327	100	105,327	100
**Murang'a**	**Kahuro**	68,418	68,418	100	68,418	100	68,418	100
**Murang'a**	**Kandara**	174,566	174,566	100	174,566	100	174,566	100
**Murang'a**	**Kangema**	117,100	116,923	100	116,923	100	116,923	100
**Murang'a**	**Kigumo**	136,752	136,700	100	136,700	100	136,700	100
**Murang'a**	**Mathioya**	99,842	99,401	100	99,401	100	99,401	100
**Nairobi**	**Dagoretti South**	260,022	259,502	100	259,502	100	259,502	100
**Nairobi**	**Langata**	271,314	264,132	97	264,132	97	264,132	97
**Nairobi**	**Kasarani**	304,905	302,586	99	302,586	99	302,586	99
**Nairobi**	**Roysambu**	258,067	257,741	100	257,741	100	257,741	100
**Nairobi**	**Westlands**	281,853	281,416	100	281,416	100	281,416	100
**Nairobi**	**Embakasi East**	370,535	370,301	100	370,301	100	370,301	100

**Nairobi**	**Embakasi South**	245,691	245,455	100	245,455	100	245,455	100
**Nairobi**	**Dagoretti North**	286,175	286,175	100	286,175	100	286,175	100
**Nairobi**	**Embakasi Central**	285,513	285,513	100	285,513	100	285,513	100
**Nairobi**	**Embakasi North**	286,819	286,819	100	286,819	100	286,819	100
**Nairobi**	**Embakasi West**	300,481	300,481	100	300,481	100	300,481	100
**Nairobi**	**Kamukunji**	267,712	267,712	100	267,712	100	267,712	100
**Nairobi**	**Kibra**	265,066	265,066	100	265,066	100	265,066	100
**Nairobi**	**Makadara**	235,028	235,028	100	235,028	100	235,028	100
**Nairobi**	**Mathare**	316,597	316,597	100	316,597	100	316,597	100
**Nairobi**	**Ruaraka**	202,127	202,127	100	202,127	100	202,127	100
**Nairobi**	**Starehe**	208,646	208,646	100	208,646	100	208,646	100
**Nakuru**	**Kuresoi North**	183,283	183,123	100	183,123	100	182,122	99
**Nakuru**	**Molo**	172,830	172,783	100	172,783	100	172,654	100
**Nakuru**	**Rongai**	186,998	185,998	99	185,998	99	185,155	99
**Nakuru**	**Subukia**	121,821	121,692	100	121,692	100	121,623	100
**Nakuru**	**Kuresoi South**	153,420	153,392	100	153,392	100	140,730	92
**Nakuru**	**Naivasha**	325,193	306,152	94	306,031	94	304,722	94
**Nakuru**	**Bahati**	203,943	203,867	100	203,867	100	203,867	100
**Nakuru**	**Gilgil**	204,621	201,427	98	201,427	98	201,396	98
**Nakuru**	**Nakuru Town East**	257,471	257,191	100	257,191	100	257,191	100
**Nakuru**	**Nakuru Town West**	200,009	199,573	100	199,573	100	199,573	100
**Nakuru**	**Njoro**	255,789	253,234	99	253,224	99	250,841	98
**Nandi**	**Chesumei**	144,537	144,474	100	144,474	100	143,980	100
**Nandi**	**Emgwen**	162,934	162,514	100	162,514	100	162,114	100
**Nandi**	**Mosop**	156,416	156,416	100	156,416	100	156,394	100
**Nandi**	**Tinderet**	123,779	122,303	99	122,279	99	121,071	98
**Nandi**	**Aldai**	185,334	184,715	100	184,715	100	183,901	99
**Nandi**	**Nandi Hills**	128,785	126,422	98	126,422	98	124,063	96
**Narok**	**Transmara East**	127,874	127,874	100	127,874	100	127,874	100
**Narok**	**Transmara West**	271,926	267,223	98	266,563	98	240,285	88
**Narok**	**Narok North**	265,190	247,488	93	247,436	93	212,841	80
**Narok**	**Narok South**	260,647	222,234	85	221,968	85	84,408	32
**Narok**	**Narok West**	190,116	163,480	86	163,374	86	93,913	49
**Narok**	**Narok East**	94,576	91,982	97	91,963	97	81,986	87
**Nyamira**	**Borabu**	80,635	80,601	100	80,601	100	80,601	100
**Nyamira**	**Nyamira North**	160,956	160,935	100	160,935	100	160,935	100
**Nyamira**	**Nyamira South**	157,408	157,408	100	157,408	100	157,408	100
**Nyamira**	**Masaba North**	108,975	108,975	100	108,975	100	108,975	100
**Nyamira**	**Manga**	91,539	91,539	100	91,539	100	91,539	100
**Nyandarua**	**Kinangop**	202,334	201,798	100	201,798	100	201,796	100
**Nyandarua**	**Ndaragwa**	93,319	92,641	99	92,641	99	92,074	99
**Nyandarua**	**Ol Joro Orok**	114,645	113,964	99	113,964	99	113,964	99
**Nyandarua**	**Kipipiri**	102,512	101,419	99	101,419	99	101,336	99
**Nyandarua**	**Ol Kalou**	126,022	125,642	100	125,642	100	125,642	100
**Nyeri**	**Mathira East**	105,873	102,430	97	102,430	97	102,430	97
**Nyeri**	**Mukurweini**	93,491	93,491	100	93,491	100	93,491	100
**Nyeri**	**Kieni East**	115,568	96,876	84	96,876	84	96,876	84
**Nyeri**	**Kieni West**	86,369	84,890	98	84,890	98	84,087	97
**Nyeri**	**Mathira West**	54,991	52,372	95	52,372	95	52,372	95
**Nyeri**	**Othaya**	96,324	95,823	99	95,823	99	95,823	99
**Nyeri**	**Tetu**	82,570	82,164	100	82,164	100	82,160	100
**Nyeri**	**Nyeri Central**	127,760	127,597	100	127,597	100	127,597	100
**Samburu**	**Samburu West**	121,401	111,906	92	111,522	92	97,308	80
**Samburu**	**Isiolo**	157,241	133,403	85	133,155	85	107,406	68
**Samburu**	**Samburu East**	84,687	58,842	69	58,798	69	35,036	41
**Samburu**	**Samburu North**	120,922	81,192	67	81,163	67	9,909	8
**Siaya**	**Alego-usonga**	225,869	222,736	99	222,736	99	221,979	98
**Siaya**	**Bondo**	184,590	166,964	90	166,964	90	163,862	89
**Siaya**	**Ugenya**	130,261	130,135	100	130,135	100	130,135	100
**Siaya**	**Rarieda**	164,552	159,024	97	159,024	97	159,024	97
**Siaya**	**Gem**	193,523	193,197	100	193,197	100	193,197	100
**Siaya**	**Ugunja**	109,933	108,568	99	108,568	99	108,568	99
**Taita Taveta**	**Taveta**	82,540	79,500	96	79,500	96	78,115	95
**Taita Taveta**	**Voi**	113,863	101,742	89	101,383	89	98,434	86
**Taita Taveta**	**Mwatate**	79,597	75,070	94	75,042	94	72,380	91
**Taita Taveta**	**Wundanyi**	72,313	70,381	97	69,744	96	65,746	91
**Tana River**	**Bura**	139,795	93,260	67	93,260	67	49,309	35
**Tana River**	**Galole**	77,977	65,054	83	64,998	83	50,598	65
**Tana River**	**Garsen**	111,477	91,951	82	89,750	81	69,322	62

**Tharaka-Nithi**	**Chuka/Igambang ombe**	137,241	136,271	99	136,271	99	136,271	99
**Tharaka-Nithi**	**Tharaka South**	77,251	76,748	99	76,748	99	73,677	95
**Tharaka-Nithi**	**Maara**	125,583	125,484	100	125,484	100	125,484	100
**Tharaka-Nithi**	**Tharaka North**	54,636	53,550	98	53,550	98	43,903	80
**Trans Nzoia**	**Endebess**	111,597	102,856	92	102,783	92	101,050	91
**Trans Nzoia**	**Kiminini**	242,545	242,513	100	242,513	100	242,513	100
**Trans Nzoia**	**Saboti**	229,837	228,252	99	228,252	99	226,209	98
**Trans Nzoia**	**Cherangany**	248,557	247,683	100	247,683	100	247,683	100
**Trans Nzoia**	**Kwanza**	180,105	180,078	100	180,078	100	180,035	100
**Turkana**	**Turkana East**	100,571	35,537	35	35,537	35	19,653	20
**Turkana**	**Turkana Central**	138,828	113,265	82	113,262	82	69,279	50
**Turkana**	**Turkana North**	148,143	83,502	56	83,502	56	6,711	5
**Turkana**	**Loima**	145,039	89,619	62	89,619	62	29,416	20
**Turkana**	**Turkana South**	145,304	110,106	76	110,101	76	57,981	40
**Turkana**	**Turkana West**	248,307	143,906	58	141,380	57	112,423	45
**Uasin Gishu**	**Ainabkoi**	139,436	139,080	100	139,080	100	139,080	100
**Uasin Gishu**	**Kesses**	199,289	198,074	99	198,074	99	196,984	99
**Uasin Gishu**	**Moiben**	179,055	178,926	100	178,926	100	178,291	100
**Uasin Gishu**	**Soy**	225,989	225,389	100	225,389	100	225,389	100
**Uasin Gishu**	**Turbo**	259,057	257,495	99	257,495	99	257,495	99
**Uasin Gishu**	**Kapseret**	202,069	201,809	100	201,809	100	201,809	100
**Vihiga**	**Emuhaya**	90,952	90,952	100	90,952	100	90,952	100
**Vihiga**	**Hamisi**	153,113	147,416	96	147,416	96	147,416	96
**Vihiga**	**Sabatia**	135,032	135,032	100	135,032	100	135,032	100
**Vihiga**	**Luanda**	104,456	104,456	100	104,456	100	104,456	100
**Vihiga**	**Vihiga**	104,962	104,962	100	104,962	100	104,962	100
**Wajir**	**Wajir South**	165,023	94,087	57	94,080	57	44,428	27
**Wajir**	**Eldas**	96,374	65,321	68	64,846	67	38,676	40
**Wajir**	**Wajir West**	102,673	67,528	66	67,514	66	36,390	35
**Wajir**	**Tarbaj**	116,715	69,791	60	67,846	58	34,724	30
**Wajir**	**Wajir East**	141,202	113,429	80	113,294	80	104,933	74
**Wajir**	**Wajir North**	183,286	109,800	60	104,724	57	50,352	27
**West Pokot**	**Central Pokot**	108,470	87,360	81	87,360	81	33,202	31
**West Pokot**	**Pokot South**	162,327	160,330	99	160,318	99	119,530	74
**West Pokot**	**West Pokot**	169,622	156,122	92	156,122	92	121,881	72
**West Pokot**	**North Pokot**	194,918	142,003	73	142,003	73	19,824	10
